# Hydrogen Peroxide Producing Titania-Silica Supraparticles as Tailorable Photocatalysts for Flow Chemistry Reactions in Microfluidic Reactors

**DOI:** 10.1021/cbe.4c00154

**Published:** 2025-01-16

**Authors:** Bettina Herbig, Egzon Cermjani, Doris Hanselmann, Angelika Schmitt, Christoph Deckers, Thomas H. Rehm, Karl Mandel, Susanne Wintzheimer

**Affiliations:** † Fraunhofer-Institute for Silicate Research ISC, Neunerplatz 2, D97082 Würzburg, Germany; ‡ Fraunhofer-Institut für Mikrotechnik und Mikrosysteme IMM, Carl-Zeiss-Str. 18-20, D55129 Mainz, Germany; § Department of Chemistry, Johannes Gutenberg-University Mainz, Duesbergweg 10-14, 55128 Mainz, Germany; ∥ Department of Chemistry and Pharmacy, Inorganic Chemistry, Friedrich-Alexander-University Erlangen-Nürnberg, Egerlandstrasse 1, D91058 Erlangen, Germany

**Keywords:** Supraparticles, photocatalyst, titania, hydrogen peroxide production, microfluidic reactor, flow chemistry, cascade reaction

## Abstract

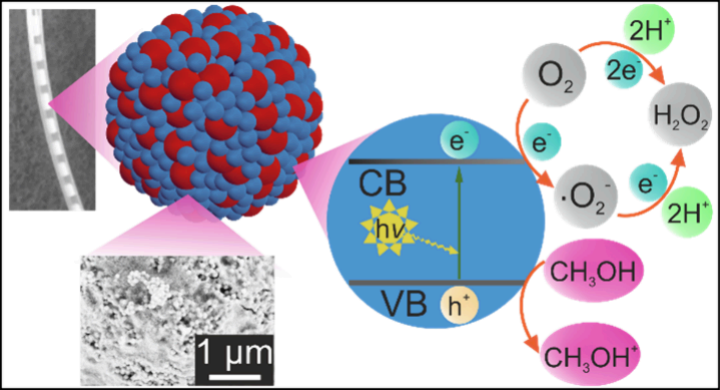

The development of hybrid catalysts for cascade reactions that demonstrate high efficiency and longevity strongly relies on the precise arrangement of the individual components within such a material. This guarantees both their proximity for enhanced interaction and, at the same time, sufficient separation avoiding mutual harm. Before the acutal design of a usually very complex hybrid catalyst, it is essential to study and understand the impact of structural characteristics on catalytic activities of each catalytically active constituent separately. This study thus focuses on a comprehensive structure–activity analysis of the component within a highly customizable TiO_2_-SiO_2_ material, which produces H_2_O_2_ photocatalytically. The tailorable design of the hybrid material is achieved through the utilization of the spray-drying process. The H_2_O_2_ productivity of the obtained so-called TiO_2_-SiO_2_ supraparticles is demonstrated in both a batch and a flow reactor, marking a crucial step toward their future application as hybrid catalysts in photoassisted cascade reactions.

## 1 Introduction

In an effort to emulate the biosynthetic efficacy observed in nature and to identify more environmentally conscious and sustainable alternatives to current chemical production methods, cascade reactions have attracted increasing attention over the past two decades.^1^ Such reactions are usually promoted by a hybrid catalyst. Prominent examples are combinations of photocatalysts and enzymes,^2^ as well as of organo- and metal catalysts.^3^ Given the incompatibility of the majority of catalysts, compartmentalization techniques, i.e., the spatial separation of the two components within a hybrid catalyst, are usually required to prevent catalyst deactivation.^1−3^ In this context, systematic studies on structure-dependent catalytic activities are highly needed, focusing not only on the often very complex, hard-to-understand complete hybrid system but also on each component on its own. Such more easily investigable model systems will pave the way to better understanding and designing future hybrid systems.

The study presented herein therefore accounts for a detailed structure–activity correlation of photocatalytic hydrogen peroxide-producing TiO_2_ nanoparticles within a highly tunable TiO_2_-silica (SiO_2_) material, which can in the future be extended by the addition of a second catalyst, such as an enzymatic or metal component. H_2_O_2_ is widely required as an oxidant in the chemical and biotechnology industries as it facilitates the oxygenation reactions of many enzymes^4^ or metal-catalyzed reactions.^5,6^ Although the anthraquinone cycle displays the predominant process for the industrial production of H_2_O_2_*ex situ*, there are multiple benefits to generating hydrogen *in situ* such as a significant process intensification and decarbonization.^6^ Photocatalytic *in situ* production of H_2_O_2_ can be facilitated by continuous flow technology and the utilization of micro reactors.^7−9^ This approach takes advantage of an enhanced multiphase reaction between oxygen and water due to increased mixing in microreactors, together with an efficient irradiation of the photocatalyst, which is enabled by a high surface-to-volume ratio. Furthermore, continuous flow processing enables a streamlined and automated steady production, excellent reaction control possibilities^10−14^ and good handling of heat development and system pressure, thereby increasing safety.^15^ In the case of capillary photoreactors utilizing fluorinated ethylene-propylene tubing (FEP), even and efficient irradiation of the reaction medium within the capillary is enabled, thereby providing optimal conditions for photocatalytic approaches.^16,17^

This study exploits for the TiO_2_–SiO_2_ material creation an approach that is ideally suited for the design of multicomponent materials with high freedom in component variations, namely, the spray-drying-assisted assembly of nanoparticles, yielding micrometer-scaled porous supraparticles.^18^ This assembly method allows for structure control and tuning, direct processing of fragile moieties such as enzymes, and subsequent post-treatments such as bio- or chemofunctionalizations, and is a fast as well as scalable process.^19^ The H_2_O_2_-productivity of the developed TiO_2_–SiO_2_ supraparticles is showcased in both batch and flow reactors as an essential step for their future use as a hybrid catalyst in photoassisted cascade reactions.

## 2 Materials and Methods

The chemicals were purchased from the following companies: tetraethoxysilane (TEOS, Sigma–Aldrich, Germany), (3-aminopropyl)triethoxysilane (Sigma-Aldrich, Germany), aqueous ammonia solution (Sigma-Aldrich, Germany), ethanol (CSC Jäkle Chemie GmbH & Co. KG, Germany), glutaraldehyde (Sigma-Aldrich, Germany, 25% in water), titanium(IV) ethoxide (Ti(OEt)_4_, 93.1%; CSC Jäkle Chemie GmbH & Co. KG, Germany), acetylacetone (2,4-pentandione, 99.3%, Sigma-Aldrich, Germany), p-toluenesulfonic acid monohydrate (Sigma-Aldrich, Germany). All chemicals were used without further purification. All syntheses were performed in deionized water.

### Nanoparticle Syntheses

TiO_2_ nanoparticles were synthesized using a hydrothermally assisted sol–gel process.^20,21^ First, an amorphous TiO_2_ precursor powder was synthesized by complexing titanium(IV) ethoxide (1.5 mol) drop by drop with acetylacetone (1 mol). Then, p-toluenesulfonic acid monohydrate (0.05 mol) was dissolved in deionized water, and the PTSA/H_2_O mixture was added to the above-mentioned solution. Without further delay, all volatiles were removed from the reaction mixture by rotational evaporation. The product is a fine yellowish powder which can easily be removed from the reaction vessel. The precursor powder is soluble in water and results in a transparent, stable sol (12 wt % TiO_2_), which was transferred into stainless steel Teflon-lined autoclaves (100 mL) and heat treated in a convection oven (Type LHT6/60/E201/OTC; Carbolite GmbH, Ubstadt-Weiher, Germany) at 160 °C at autogenous pressure for 4 h. The autoclaves were taken out of the hot oven and cooled down to room temperature before opening them. The products obtained were of gel-like consistency and showed brownish-red color. The gels were removed from the liners, and a dispersion was prepared by redispersing the gels containing nanocrystalline TiO_2_ particles in water for spray-drying experiments.

SiO_2_ nanoparticles with a diameter of 80 nm were synthesized using a recently published method that is based on the Stöber process.^22^ In a typical experiment, 300 mL of ethanol was mixed with 15 mL of aqueous ammonia solution (25 wt %). The mixture was vigorously stirred while 15 g (0.072 mol) of TEOS was added and subsequently stirred overnight. To purify the nanoparticles, the ethanol/ammonia solution was removed by rotary evaporation, and the mixture was dialyzed for 24 h with multiple water changes using a dialysis membrane with a molecular weight cutoff of 10 kDa (cellulose hydrate membrane, Nadir-dialysis tubing, Roth, Germany). The functionalization of the SiO_2_ nanoparticles with (3-aminopropyl)triethoxysilane and glutaric acid was conducted following the protocol described elsewhere and the functionalized nanoparticles were subsequently redispersed in water.^23^

### Supraparticle Fabrications

Either plain TiO_2_ or mixed TiO_2_–SiO_2_ nanoparticle dispersions at weight ratios of 1:6, 1:3, 1:2, 1:1, 2:1, 3:1, and 6:1 were spray-dried using a B-290 mini spray-dryer (Büchi, Switzerland). Upon mixing the TiO_2_ with the SiO_2_ dispersion, the obtained binary nanoparticle dispersion displays pH values around 5 due to the high acidity of the TiO_2_ precursor solutions (yielding core–shell structures upon spray-drying due to electrostatically stabilized nanoparticles, as mentioned in the Results and Discussion). For the creation of intermixed supraparticles, the pH value of the precursor dispersion was adjusted to a pH of 7.5 with the dropwise addition of 25% ammonia solution while stirring the dispersion (for a destabilization and preagglomeration of the nanoparticles). The spray-dryer was equipped with a two-fluid nozzle, and the inlet temperature was set at 30 °C, with an outlet temperature of about 24 °C at 1 mL/min velocity.

### Characterization Methods

The obtained SiO_2_ and TiO_2_ nanoparticles were characterized as described in SI. The spray-dried supraparticles were characterized using scanning electron microscopy (SEM, Zeiss Supra 25, Germany). The acceleration voltage for SEM was 2 kV. To prepare cross section samples, 10 mg of supraparticles was dispersed in 500 μL of lacquer. The mixture was then cured at 100 °C and cut using a scalpel. These samples were fixed onto a sample holder using conductive silver and sputtered with platinum for 5 s at 50 mA using the MED 010 instrument from Balzers Union. Laser diffraction (Microtrak Bluewave) was used to measure the supraparticle size distributions. Further supraparticle characterization methods (Nitrogen sorption and SEM-EDS) are listed in SI.

#### Photocatalytic Experiments in Batch Mode

The amount of photocatalytically produced hydrogen peroxide was determined by using the Eisenberg method.^24^ Here 0.125 g of plain TiO_2_ or TiO_2_–SiO_2_ supraparticles was suspended in 250 mL deionized water and stirred at 375 rpm and 25 °C in an in-house built reactor (see Figure S1a,b). Air was continuously bubbled in (250 l/h), and irradiation was performed through a borosilicate glass window on one side of the reactor by adjusting four 365 nm LEDs (Nichia NCSU276A UV SMD-LED, 780 mW) in front. Before irradiation (t = 0 min) and after different irradiation times (5 to 20 min), an aliquot of the solution was removed from the reactor. To 5 g of the solution, 0.5 mL of TiOSO_4_ solution (1.9–2.1%, Sigma-Aldrich) was added and filtered through a 0.2 μm syringe filter. The resulting yellow-colored titanium peroxo-complex was analyzed photometrically using a Specord 50, Analytik Jena GmbH & Co.KG spectrophotometer. The absorption at 408 nm was chosen to plot a calibration curve using standard H_2_O_2_ concentrations (20 to 1000 μmol/L, via dilution of a 30% hydrogen peroxide solution, Carl Roth) and to estimate the concentrations of photocatalytically produced H_2_O_2_. The obtained calibration curve is shown in Figure S1c (in the Supporting Information). The error bars in the catalytic tests are obtained via performing 3 reproductions of both the test and the material preparation.

#### Photocatalytic Experiments in Continuous-Flow Mode

A capillary photoreactor was used for continuous flow synthesis of H_2_O_2_ (Figure S2, Table S1). The LED array (Table S2, Figure S3) is cooled by water to approximately 10 °C, and the reactor setup is equipped with an additional thermostat, which maintains the desired temperature within the reactor. The reaction suspension is introduced into the reactor via syringe pumps, which are equipped with an integrated external magnetic stirring motor, thereby ensuring an even and continuous agitation of the aqueous suspension within the syringe. A gas bottle containing synthetic air and a mass flow controller for providing oxygen as a reactant and setting precise gas flow rates are connected to the system by a three-way valve (PTFE, 1.6 mm bore). The supraparticles are dispersed in water and subsequently transferred to a syringe. Subsequently, the suspension is fed into the capillary photoreactor under constant stirring at specified flow rates (Tables S3–S5). The reaction is initiated by irradiating the reaction suspension at a wavelength of 365 nm for varying periods of time, thereby providing data for time-dependent generation of H_2_O_2_. Subsequently, the suspension is collected, and the concentration of H_2_O_2_ is determined following the filtration of the supraparticles and applying the Eisenberg method.^24^

## 3 Results and Discussion

**Figure 1:**
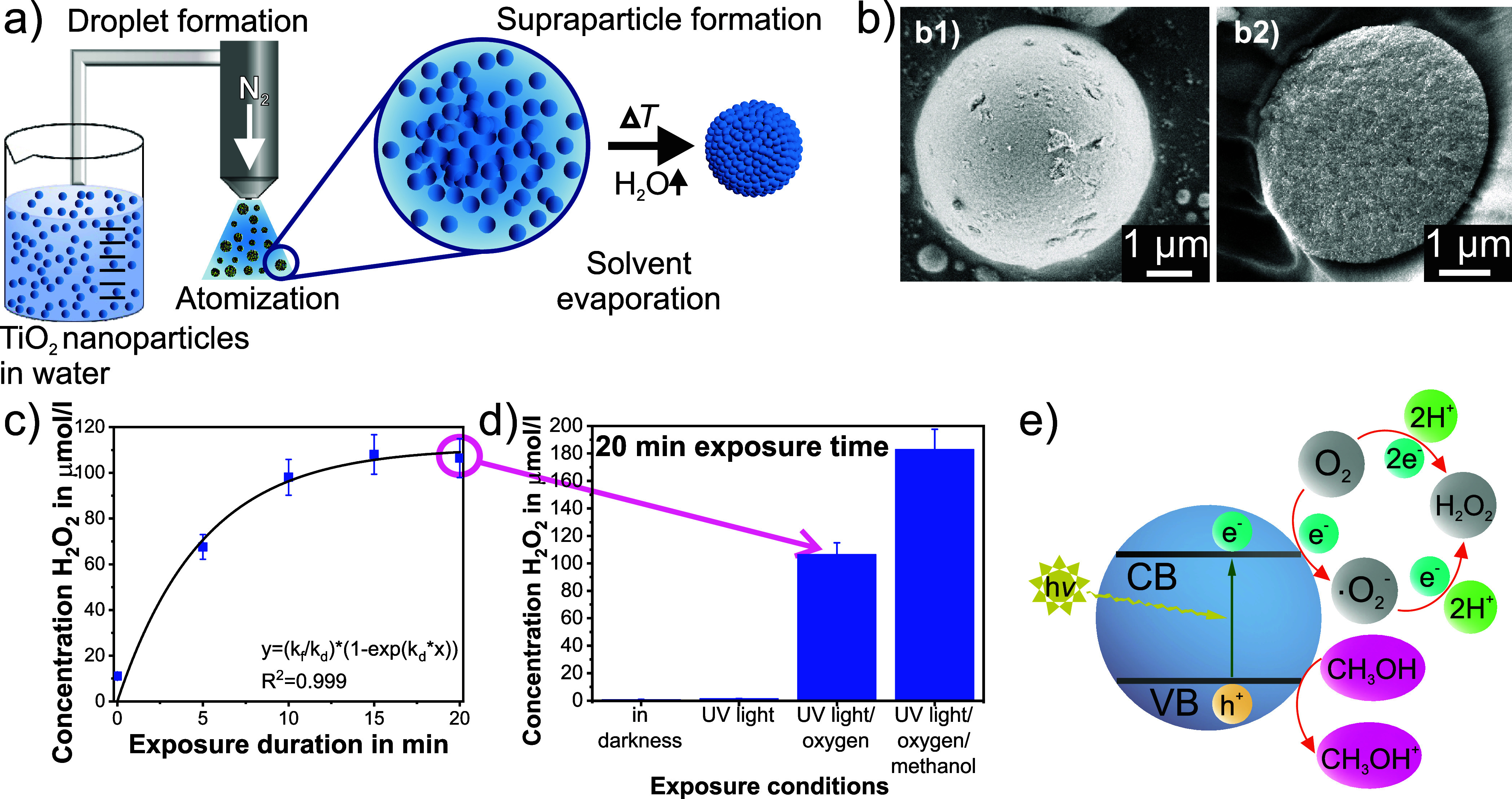
(a) Schematic representation of the supraparticle fabrication during spray-drying via droplet evaporation using TiO_2_ nanoparticles dispersed in water as precursors (adapted from Reichstein et al.^28^ under terms of the CC-BY license). (b) SEM micrographs of (b1) a TiO_2_ supraparticle and (b2) its cross section. (c) Cumulated concentration of H_2_O_2_ produced by TiO_2_ supraparticles throughout UV-light exposure in the presence of oxygen, fitted with the kinetic equation of the photocatalytic H_2_O_2_ production y = *k*_f_/*k*_d_*(1 – exp(−*k*_d_*x*) with *k*_f_ and *k*_d_ being the formation and decomposition rate constants and *x* being the exposure time,^29^ and (d) cumulated concentration of H_2_O_2_ produced by TiO_2_ supraparticles either after stirring in darkness, exposure to UV-light, exposure to UV-light and oxygen, as well as exposure to UV-light, oxygen, and methanol (1.0 vol %) for 20 min. (e) Schematic representation of the photocatalytic H_2_O_2_ production using a TiO_2_ nanoparticle as the photocatalyst.

The synthesized TiO_2_ nanoparticles utilized for assembling H_2_O_2_-producing supraparticles via spray-drying, provide sizes of around 7 nm, anatase phase, and a charged surface with its isoelectric point around pH 7 (determined via transmission electron microscopy, dynamic light scattering, X-ray diffraction, and zeta potential measurements; Figure S4 in the Supporting Information and studied in detail elsewhere^20,21,25−27^). The process of spray-drying a TiO_2_ nanoparticle dispersion is depicted in Figure 1a. Initially, the dispersion is pumped through a nozzle, resulting in the release of a fine mist into a heated chamber. In this chamber, the solvent present in the individual droplets is evaporated, forcing the contained nanoparticles together to form supraparticles that can be retrieved as a powder sample.

The spherical, dense morphology of the obtained TiO_2_ supraparticles is shown in scanning electron micrographs of Figure 1b and Figure S5a (in the Supporting Information). They possess micrometer sizes in the range of 0.6 to 5 μm (determined via a laser diffraction measurement; Figure S5b, Supporting Information). To investigate the textural properties of the TiO_2_ supraparticles, an N_2_ sorption measurement was performed at 77 K (Figure S5c to e, Supporting Information). The obtained type IV isotherm (IUPAC classification) is typical of a mesoporous material. The pore size distribution (PSD) and cumulative pore volume obtained by applying nonlocal density functional theory (NLDFT) to the adsorption branches indicate an average pore size of around 5 nm. The surface area of the TiO_2_ supraparticles calculated using the Brunauer–Emmett–Teller (BET) model is 180 m^2^/g.

The H_2_O_2_-productivity of the TiO_2_ supraparticles was determined in a catalytic test setup based on a batch reactor, a UV light source, and a pressurized air inlet (see Materials and Methods and Figure S1a and b in the Supporting Information). The quantification of H_2_O_2_ was achieved using the Eisenberg method,^24^ which is based on the light absorbance at 408 nm of H_2_(TiO_2_(SO_4_)_2_) formed by the reaction of H_2_O_2_ with TiOSO_4_ and H_2_SO_4_. The obtained calibration curve is shown in Figure S1c (in the Supporting Information). The determined concentration of H_2_O_2_ over the UV light exposure duration is displayed in Figure 1c. After an initial almost continuous increase in H_2_O_2_, its production reaches a steady state after approximately 15 min of irradiation time (which will be discussed later on). In this context, it has to be mentioned that a significant amount of H_2_O_2_ may also adsorb on the (inner and outer) surface of the TiO_2_ supraparticles (Supporting Information Figure S6) and is thus not available in the solution and consequently is not quantifiable via the utilized method. As the H_2_O_2_ production rate is highly suppressed by removing O_2_ or UV light irradiation (Figure 1d), it can be concluded that the photocatalytic O_2_ reduction is the dominant route for H_2_O_2_ generation over TiO_2_ supraparticles (Figure 1e). Their productivity can be further enhanced by adding a hole scavenger like methanol to the reaction (Figure 1d and Figure S7) which decreases the electron–hole recombination probability as well as the undesired oxidation of H_2_O_2_.^30,31^ Due to the continuous air inlet, the O_2_ concentration is considered to be constant. Consequently, the formation rate of H_2_O_2_ can be considered as zero-order kinetic.^29,32^ At the same time, H_2_O_2_ as a metastable molecule undergoes photodegradation such as reduction to water and oxidation to O_2_ (the latter can partly be suppressed by the addition of methanol). Additionally, H_2_O_2_ is easily converted to Ti-OOH complexes when it is in touch with the Ti–OH groups on the TiO_2_ surface. Subsequently, Ti-OOH is once again reduced upon reaction with an electron (Ti-OOH + H^+^ + e^–^ → Ti–OH + ·OH) competing with the oxygen reduction reaction.^33−35^ These degradation processes presumably follow first-order kinetics related to the concentration of H_2_O_2_. Consequently, the total H_2_O_2_ generation *y* should follow the kinetic equation y = *k*_f_/*k*_d_*(1 – exp(−*k*_d_*x*) with *k*_f_ and *k*_d_ being the formation and decomposition rate constants and *x* being the exposure time.^29^ Under these conditions, the concentration of H_2_O_2_ will reach a steady state after a certain amount of irradiation. The recorded data (Figure 1c) can be fitted very well by this equation (*R*^2^ = 0.999). In general, TiO_2_ is often claimed to be an inefficient photocatalyst for H_2_O_2_ mainly due to a high electron–hole recombination rate and a high decomposition rate of H_2_O_2_ on TiO_2_, especially on the commercial benchmark catalyst P25.^31,36,37^ Indeed, we also observed negligible activity of commercial P25 and P90 catalysts in our catalytic batch reactor setup (Figure S8 in the Supporting Information). The significantly higher activity of the herein-developed TiO_2_ supraparticles can potentially be attributed to the wet-chemical nanoparticle synthesis (compared to flame-pyrolyzed P25 and P90). Organic molecules from the synthesis, such as acetylacetonate (and its decomposition product acetate), may still be adsorbed on the TiO_2_ surface and consequently decrease the rate of photodegradation of H_2_O_2_. This may be attributed to either a steric effect in which these molecules block the sites where H_2_O_2_ is degraded or their efficient competition with H_2_O_2_ for valence band holes.^32^ Another reason for a significantly higher activity of the developed supraparticles compared to P25 as well as P90 could be the presence of mixed anatase–rutile phases in the commercial sample compared to pure anatase in the supraparticles.^38^ Rutile is attributed to decompose H_2_O_2_ to a higher extent than anatase due to a different reaction mechanism (on rutile H_2_O_2_ reduction is dominant, on anatase its oxidation).^38^ In general, the obtained H_2_O_2_ productivity of the TiO_2_ supraparticles lies within the expected broad activity range of photocatalytic TiO_2_ materials (Table S6).

While the H_2_O_2_ productivity of the synthesized TiO_2_ supraparticles has been confirmed, for the envisaged aim to develop a dual-component particle for future photoassisted cascade reactions, the introduction of a second, inert material is crucial. Such material is not meant to interfere with the light interaction of TiO_2_ or provide energy transfers between the two materials but serves for the immobilization of enzymes or organocatalysts to create hybrid catalysts and allows for spatial separation of these components from the photocatalyst to avoid, for example, their photodeactivation. The addition of a second component, e.g., functionalized SiO_2_ nanoparticles, comes along with the challenge of directly influencing the distribution of TiO_2_ nanoparticles within a supraparticle. This affects the interaction of the photocatalyst component with UV light and, consequently, the H_2_O_2_ productivity of the system. This structure–reactivity correlation for the photocatalyst component within a supraparticle is the main focus of the hereafter-described study.

**Figure 2:**
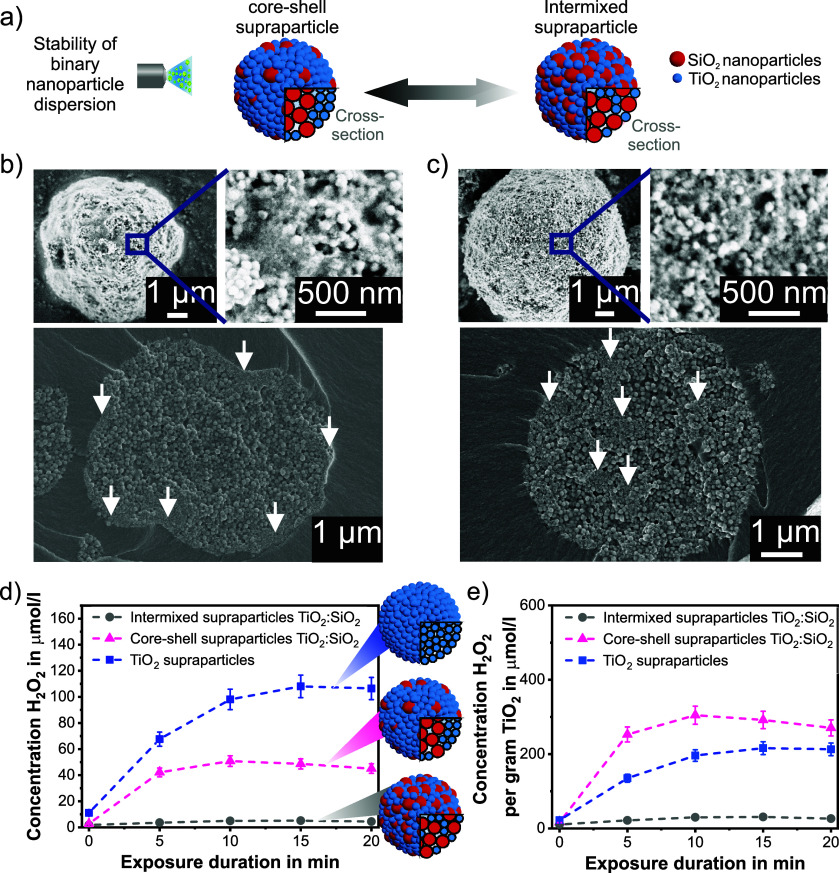
(a) Scheme showing the fabrication of either core–shell or intermixed supraparticles dependent on the stability of the TiO_2_–SiO_2_ nanoparticle precursor dispersion (displaying a TiO_2_ to SiO_2_ weight ratio of 1:2). Stable dispersions yield core–shell supraparticles, while destabilized dispersions result in intermixed supraparticles. SEM micrographs of (b) a core–shell supraparticle: its surface and its cross section and (c) an intermixed supraparticle: its surface and its cross section. White arrows indicate the areas of increased TiO_2_ nanoparticle accumulation within the supraparticle cross sections. (d) Cumulated concentration of H_2_O_2_ produced by TiO_2_, as well as TiO_2_–SiO_2_ core–shell and intermixed supraparticles over the UV-light exposure duration (dashed lines are only for guiding the eye), and (e) the same values referenced to the weight of contained photocatalyst within each supraparticle sample (dashed lines are only for guiding the eye).

As the second nanoparticle building block, glutaraldehyde-modified SiO_2_ nanoparticles ∼100 nm in size were chosen (see Figure S9 in the Supporting Information for SiO_2_ nanoparticle characterization). SiO_2_ nanoparticles are probably the most frequently used material for the creation of hybrid catalysts as they have been proven to provide outstanding properties required for the immobilization of catalysts, such as high surface area and inertness. Additionally, SiO_2_ can be easily organo-functionalized (e.g., with glutaraldehyde) to control interactions between enzymes or organocatalysts and support surface and to ultimately yield a covalent attachment without catalyst deactivation.^39−43^ Using a binary (aqueous) dispersion of TiO_2_ and SiO_2_ nanoparticles with a fixed TiO_2_:SiO_2_ weight ratio of 1:2, the spray-drying-assisted assembly can either yield a core–shell or an intermixed arrangement of both components (Figure 2a) dependent on the dispersion stability and electrostatic surface charges of the precursor nanoparticles.^25,44^ Below pH values of 6, both nanoparticles are positively charged and form an excellently stabilized dispersion due to mutual electrostatic repulsions. This results in a core–shell structure upon spray-drying, where the larger (SiO_2_) nanoparticles display the core and the smaller (TiO_2_) ones display the shell of a supraparticle, as the forces acting on the nanoparticles during solvent evaporation depend on their sizes. The evaporation of the solvent at the air/liquid interface causes a drag force on the nanoparticles toward the interface. Small nanoparticles migrate more easily and quickly toward the interface, while larger nanoparticles tend to remain in the droplet center. Upon destabilization of the dispersion due to a pH value around 7 to 8, which approximates the point-of-zero-charge of the nanoparticles (see zeta potential measurements of both nanoparticle types in Figures S4d and S9b), both nanoparticle types already preagglomerate statistically in the dispersion before spray-drying. This results in an intermixed supraparticle composition where both nanoparticle types are more or less homogeneously distributed throughout the supraparticle. The obtained samples indeed display the envisaged core–shell and intermixed structures as shown in scanning electron micrographs (Figure 2b, c and Figure S10 in the Supporting Information). While they both provide mean diameters similar to those of the pure TiO_2_ supraparticles (Figure S11a), their isotherms obtained in nitrogen sorption measurements show an abrupt and sharp hysteresis at p·p_0_^–1^ close to 1 without plateau, indicating the presence of larger meso- and macropores whose complete pore filling cannot entirely be resolved by adsorption, which is also indicated by the PSD and the cumulative pore volume. The intermixed supraparticles display a higher amount of mesopores than the core–shell ones (as derived from the PSD and cumulative pore volume plots). The core–shell structure provides a higher BET surface area of 145 m^2^/g compared to that of 102 m^2^/g of the intermixed TiO_2_–SiO_2_ supraparticles. Consequently, despite the same amount of TiO_2_ and SiO_2_ nanoparticles within both samples, their differing internal structure significantly affects their accessible surface area and their cumulative pore volume.

Regarding the H_2_O_2_ productivity of these TiO_2_–SiO_2_ supraparticles, it was expected that the introduction of the inert SiO_2_ component would decrease the overall activity of the catalyst (Figure 2d). However, the extent of the decline in catalytic activity is strongly dependent on the position of the TiO_2_ nanoparticles within a supraparticle. The core–shell supraparticles produce significantly higher H_2_O_2_ amounts compared to the intermixed sample, which is due to a higher number of TiO_2_ nanoparticles located at the outside of the supraparticles. They can thus directly interact with the UV light that approaches the supraparticle^45^ or that is reflected from the inner SiO_2_-based core of the supraparticle. In the case of intermixed supraparticles, TiO_2_ presumably cannot be approached by UV irradiation due to the strong scattering of light by SiO_2_ nanoparticles. If the H_2_O_2_ productivity is displayed as per gram of photocatalyst component contained in a supraparticle sample (Figure 2e), this phenomenon becomes even more evident. TiO_2_ nanoparticles located in the outer shell of a supraparticle are more involved in photocatalysis compared with photocatalyst nanoparticles in the supraparticle core. Thus, the TiO_2_–SiO_2_ core–shell sample provides even higher activity per gram of contained TiO_2_ than pure TiO_2_ supraparticles. In this context, it has to be mentioned that the H_2_O_2_ formation pathway is not affected by the SiO_2_ component due to its amorphous/inert nature.

**Figure 3:**
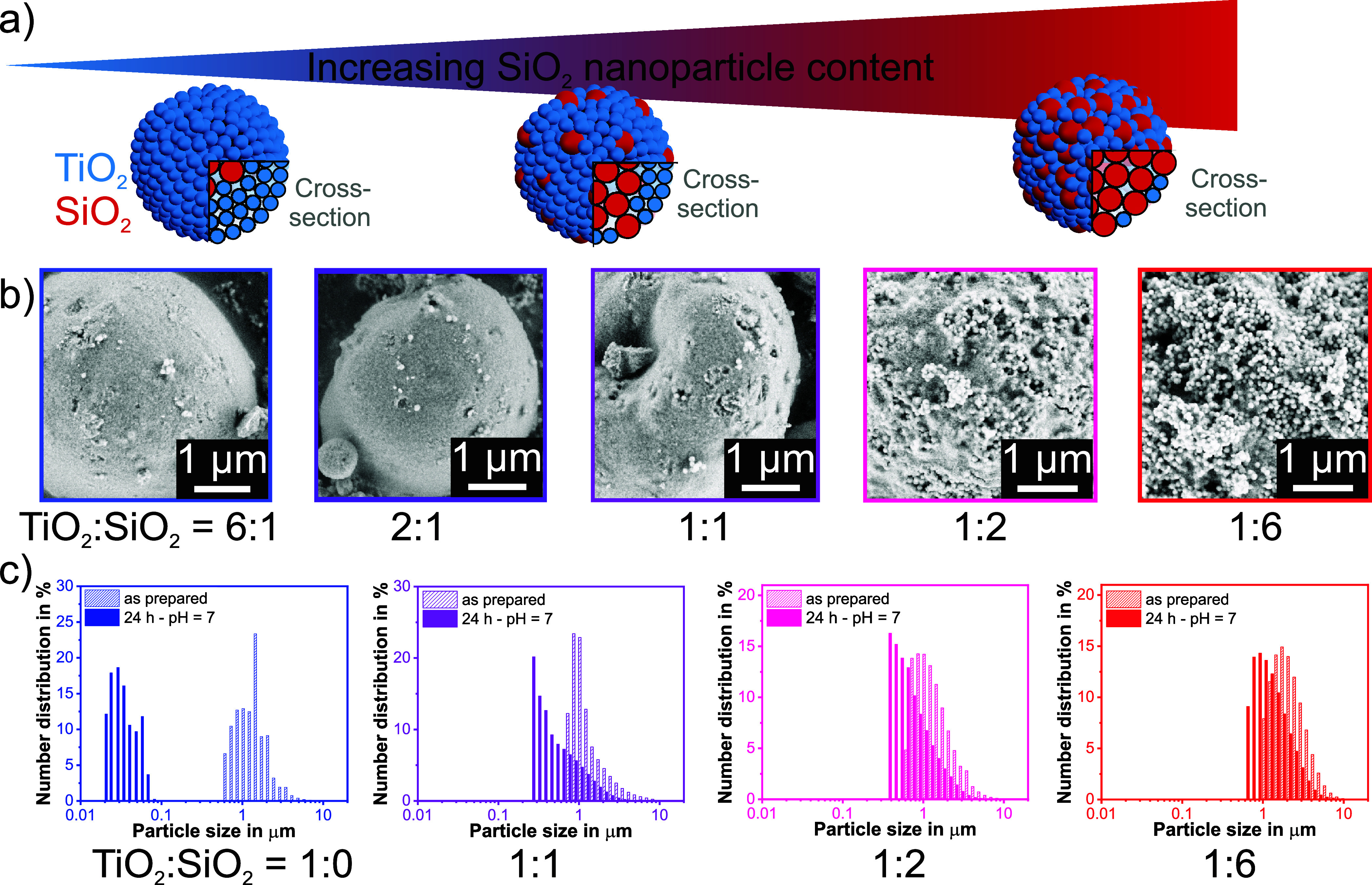
(a) Scheme showing the surface composition of core–shell supraparticles dependent on the TiO_2_ to SiO_2_ weight ratio. An increase in the SiO_2_ nanoparticle content leads to their increased presence on the supraparticle surface. (b) SEM micrographs of core–shell supraparticles with increasing SiO_2_ nanoparticle content, i.e. rising from a TiO_2_:SiO_2_ weight ratio of 6:1 to 1:6. (c) Number-weighted particle size distribution of TiO_2_ supraparticles and core–shell supraparticles with increasing SiO_2_ nanoparticle contents, i.e., rising from a TiO_2_ to SiO_2_ weight ratio of 1:1 to 1:6; measured as prepared and after strong stirring (at 250 rpm) as a suspension in water for 24 h (pH = 7, using a magnetic stirring bar).

After the core–shell supraparticle structure was identified as the most preferred option, the determination of the most appropriate (weight) ratio of TiO_2_ to SiO_2_ within a supraparticle was of high importance for designing the most appropriate catalyst structure. Supraparticles were thus synthesized with varying TiO_2_ to SiO_2_ contents ranging from 6:1 to 1:6 TiO_2_:SiO_2_ as displayed in Figure 3a. The obtained supraparticle structures are shown in Figure 3b and Figure S12 (in the Supporting Information). With increasing SiO_2_ content, TiO_2_ shell thickness steadily decreases until a complete coverage of the SiO_2_ core is no longer given and single SiO_2_ nanoparticles in the case of TiO_2_:SiO_2_ = 1:1 or even clusters of SiO_2_ in the case of TiO_2_:SiO_2_ = 1:2, 1:3 or 1:6 stick out of the TiO_2_ shell and create a rougher (outer) supraparticle surface. With increasing SiO_2_, the supraparticle diameters slightly increase (Figure S13a in the Supporting Information) and the supraparticles display a higher amount of macro and a lower amount of mesopores (as derived from the nitrogen sorption isotherms, the PSD, and the cumulative pore volume plots, Figure S13b and c in the Supporting Information). BET surface area continuously decreases from 169 to 64 m^2^/g for supraparticles with TiO_2_:SiO_2_ ratios of 6:1 to 1:6.

As for the application of such supraparticles in batch and flow reactors, their mechanical stability is a crucial requirement, and the stability of the obtained supraparticle samples was examined by measuring their particle size distribution before and after stirring as a suspension in water for 24 h (Figure 3c and Figure S14 in the Supporting Information). It is evident that with increasing SiO_2_ nanoparticle content, the stability of the supraparticles significantly increases as the original supraparticle diameters are retained after 24 h of stirring in suspension. A small decrease in diameters after 24 h of stirring as detected for pure SiO_2_ supraparticles (Figure S14 in the Supporting Information) and supraparticles with TiO_2_:SiO_2_ weight ratios of 1:2 and 1:6 is attributed to the separation of small agglomerates of supraparticles into single supraparticles. The stabilization effect is attributed to the ability of glutar aldehyde to self-polymerize^46^ and of SiO_2_ to form Si–O–Si bridges between nanoparticles during spray-drying,^47^ which presumably also stabilizes the contained TiO_2_ nanoparticles within the supraparticle shell.

**Figure 4:**
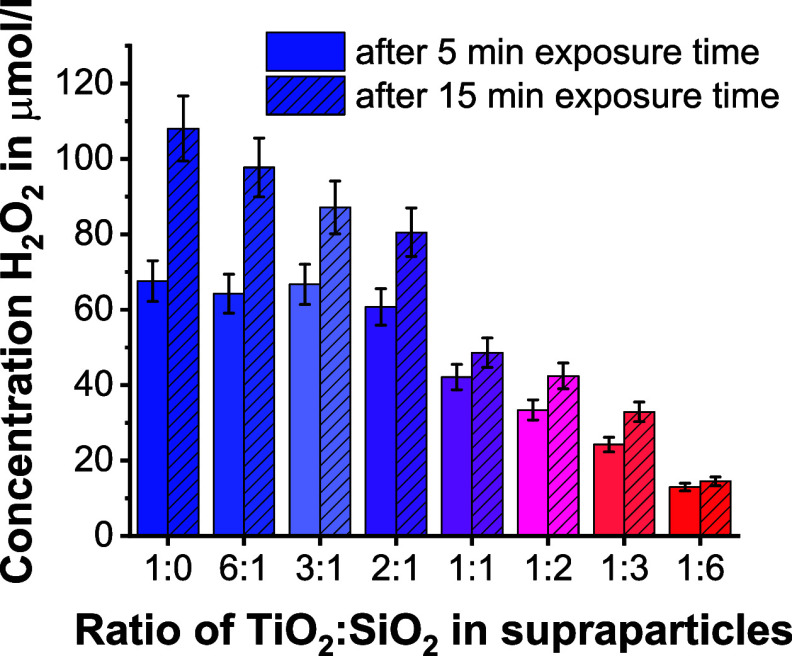
Cumulated concentration of H_2_O_2_ after 5 or 15 min of UV-light exposure produced by core–shell supraparticles with increasing SiO_2_ nanoparticle contents, i.e., rising from a TiO_2_ to SiO_2_ weight ratio of 6:1 to 1:6, in comparison to pure TiO_2_ supraparticles (1:0 TiO_2_:SiO_2_ ratio).

The H_2_O_2_ productivity of these samples after 5 and 15 min of UV light irradiation is displayed in Figure 4 and Figure S15. In general, without considering the internal structure of the supraparticle, a continuous decrease in activity is expected with increasing SiO_2_ and thus decreasing TiO_2_ contents. Supraparticles with a TiO_2_–SiO_2_ ratio of 6:1 up to 2:1 however provide approximately the same H_2_O_2_ productivity after a 5 min exposure time. At this point, the samples have not reached their steady state, and the reaction follows a first-order kinetic as H_2_O_2_ production is dominant while its decomposition does not yet play a significant role in the overall reaction. It is expected that for the efficient oxygen reduction to H_2_O_2_ a high interaction of the photocatalyst with UV light is crucial. This is why only the TiO_2_ nanoparticles located in the outer shell of a supraparticle participate in this reaction, while the ones located inside a supraparticle do not play a role in it due to no interaction with UV light (as already mentioned before). The composition of the outer shell of the supraparticles stays the same for all supraparticles with a TiO_2_–SiO_2_ ratio of 6:1 up to 2:1, i.e. displays exclusively TiO_2_ nanoparticles. This consequently explains why they all provide the same concentration of produced H_2_O_2_ after a 5 min exposure time.

Regarding the H_2_O_2_ concentration after 15 min of irradiation time, the supraparticle samples with a TiO_2_–SiO_2_ ratio of 6:1 up to 2:1 now show the expected decreasing activity with declining TiO_2_ content within a supraparticle sample. In this context, it must be mentioned that all samples have reached their steady state in H_2_O_2_ production at this point in time. It is assumed that for the steady-state concentration, the overall amount of present TiO_2_ catalyst and thus catalytically active surface area combined with the local light intensity plays a significant role.^31^ Additionally, the porous structure of the supraparticle enables adsorption, desorption, and diffusion processes of reactant and product molecules throughout the entire supraparticle.^48,49^ The studied samples significantly differ in the total amount of surface area, the macro- and mesopore volumes, and the overall pore volumes. All these parameters may also affect the steady-state concentration of the samples (as already observed for other reactions with supraparticle catalysts^50^).

In the case of supraparticles with a TiO_2_–SiO_2_ ratio of 1:1 up to 1:6, the shell composition changes continuously, i.e., the amount of TiO_2_ in the outer shell(s) decreases, with increasing SiO_2_ amount while no further TiO_2_ nanoparticles are enclosed inside the supraparticle. This results in the expected decreasing activity with declining TiO_2_ content both after 5 and 15 min, while it has to be considered that the reactions have reached steady state already at 5 min irradiation time for these samples. At this state, other supraparticle properties such as the overall amount of the photocatalyst component, mesopore-macropore concentrations, or the overall surface area of a supraparticle, seem to play in general a more dominant role than the supraparticle structure itself (as discussed before). The evaluations of both the mechanical stability and the H_2_O_2_ productivity of the produced supraparticles and their correlation to the TiO_2_:SiO_2_ nanoparticle ratios as well as the overall supraparticle structures showed that both parameters evolve in opposite ways. Consequently, the supraparticle sample with a TiO_2_–SiO_2_ ratio of 1:2 appears to be the most promising candidate for use as a future hybrid catalyst. It is expected to provide sufficient mechanical stability and adequate H_2_O_2_ productivity. Regarding the impact of the supraparticle structure on a potential postsynthetic grafting of an enzyme or organocatalyst to the silica nanoparticle components, the TiO_2_–SiO_2_ ratio of 1:2 is also expected to be favorable. While the TiO_2_-based supraparticles display relatively narrow pore sizes (especially for the presented core–shell compositions) which potentially hinder the diffusion of large organic or biological molecules into the center of the supraparticles, the interruption of the outer TiO_2_ shell of a supraparticle by zones of silica nanoparticles as shown in Figure 3 ensures an accessible surface for the successful grafting of such large molecules. Additionally, these silica nanoparticle “islands” on the outer supraparticle surface may also allow a certain spatial separation of an attached organo- or biocatalyst from the photocatalytically active nanoparticles.

Regarding the stability and reusability of the studied supraparticles, several additional experiments were conducted. On the one hand, it was determined if both TiO_2_ and TiO_2_–SiO_2_ (1:2 ratio) supraparticles retain their H_2_O_2_ productivity after 24 h of stirring (Figure S16), which indeed was the case. The partial destruction of the TiO_2_ supraparticles upon stirring for 24 h (Figure 3c) seemed to only slightly affect the overall productivity of this sample, while the TiO_2_–SiO_2_ conserved as expected their activity due to no structural supraparticle changes. On the other hand, the morphology, chemical composition, and productivity of these catalyst supraparticles were conserved after a photocatalytic batch run (for more details, see Figure S17 and the corresponding discussion).

**Figure 5:**
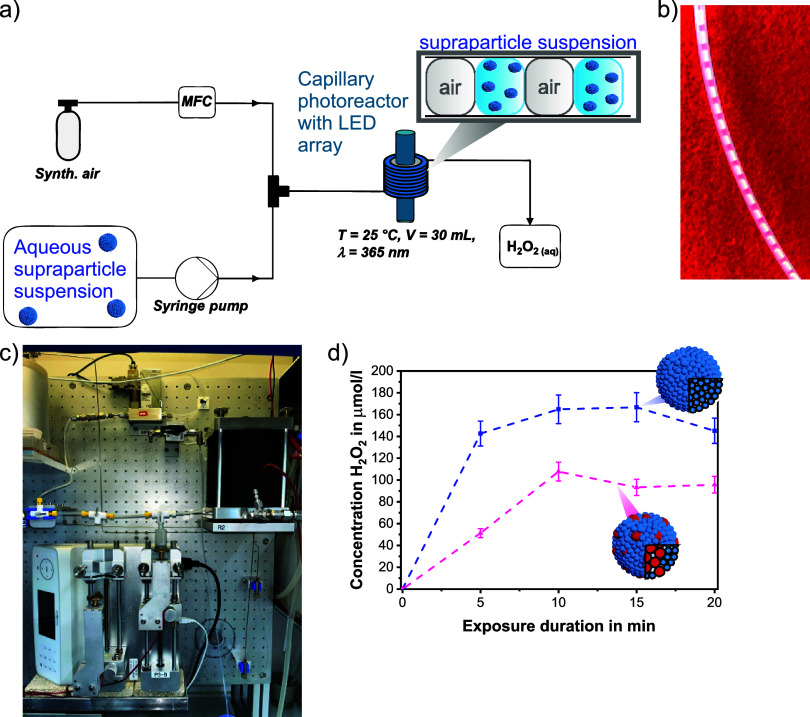
(a) Scheme for the photocatalytic generation of H_2_O_2_ in continuous flow mode and (b) photograph of the slug flow consisting of air segments and segments of aqueous TiO_2_/SiO_2_ (1:2) suspension. (c) Photograph of the continuous flow photoreactor with syringe pumps illustrating the main plant setup and (d) cumulated concentration of H_2_O_2_ produced by TiO_2_, as well as TiO_2_–SiO_2_ (1:2) core–shell supraparticles in the continuous flow setup over the UV-light exposure duration (dashed lines are only for guiding the eye).

While the first study of H_2_O_2_-producing supraparticles was conducted in batch reactions, for further upscaling, reactions involving gases such as oxygen are severely limited in batch due to mass transfer limitations and safety concerns when high system pressures are applied.^16^ The use of light for photochemical reactions is similarly complicated in batches due to the large reactor sizes and difficulties regarding efficient irradiation of reaction suspension.^16^ This is why this study aimed to achieve the continuous flow synthesis of H_2_O_2_ through the use of the developed supraparticles with a capillary photoreactor comprising FEP tubing and a centrally positioned LED array for the irradiation of the reaction suspension (Figure 5a and Materials and Methods section). Synthetic air was introduced into the capillary reactor, together with the aqueous particle suspension, to create a three-phase slug flow (Figure 5b and c). The continuous flow movement of the suspension enabled by the slug flow ensured an evenly distributed catalyst for the reaction and prevented clogging.^51^

The H_2_O_2_ production by using TiO_2_ and TiO_2_-SiO_2_ supraparticles with a 1:2 weight ratio was investigated in the capillary photoreactor (Figure 5d) using similar amounts of photocatalyst material as in batch (Table S3). The equilibrium between the production and degradation of H_2_O_2_ on the surface of TiO_2_ (observed as a plateau) was achieved at a residence time of 10 min, due to the degradation of H_2_O_2_ on the TiO_2_ surface at higher concentrations. Once again, a decrease in H_2_O_2_ generation was observed with a lower fraction of photocatalyst components, while the supraparticle mass fraction was kept constant. The overall achieved H_2_O_2_ concentrations were significantly higher in continuous flow compared to those in the initial batch experiments. In this context, it also must be mentioned that the supraparticles maintained their stability throughout the continuous flow reaction (Figure S18).

**Figure 6:**
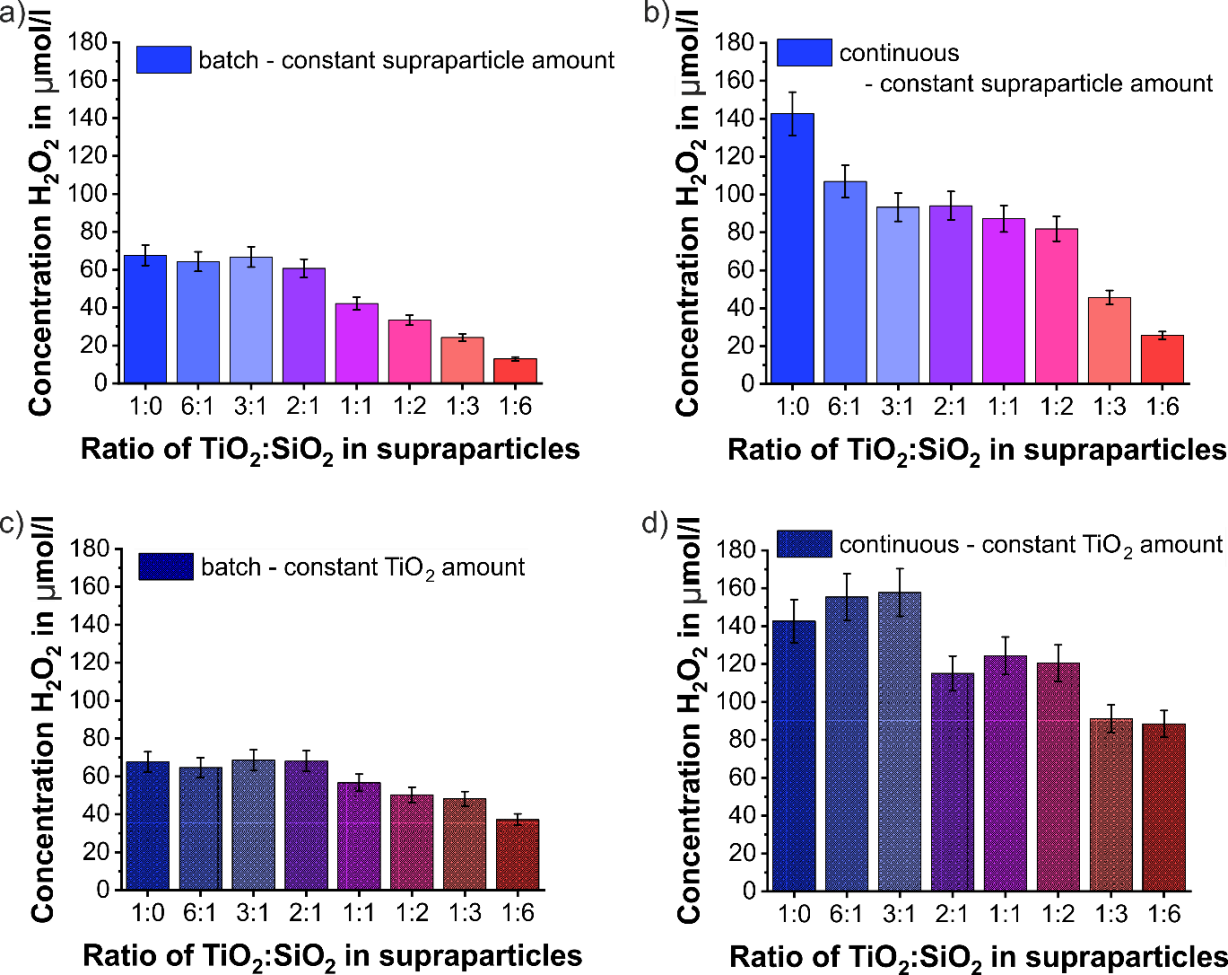
Cumulated concentration of H_2_O_2_ after 5 min UV-light exposure produced by core–shell supraparticles with increasing SiO_2_ nanoparticle contents, i.e., rising from a TiO_2_ to SiO_2_ weight ratio of 6:1 to 1:6, in comparison to pure TiO_2_ supraparticles (1:0 TiO_2_:SiO_2_ ratio): obtained in (a) batch reactions and (b) continuous flow reactions keeping the total supraparticle amount per batch constant, as well as in (c) batch reactions and (d) continuous flow reactions keeping the total TiO_2_ photocatalyst amount per batch constant.

For obtaining further insights, the concentrations of produced H_2_O_2_ after 5 min of reaction time are compared in the following of both batch and flow synthesis for all supraparticle samples with TiO_2_:SiO_2_ weight ratios of 6:1 to 1:6 and pure TiO_2_ supraparticles either with the same amount of supraparticles (Figure 6a and b, as well as Figure S19a, b) or with the same amount of the photocatalytically active TiO_2_ component (Figure 6c and d, as well as Figure S19c, d) in each reaction (Table S4 and S5). Once again, it is evident that the overall achieved H_2_O_2_ concentrations are significantly higher in continuous flow compared to the batch experiments (especially, upon referencing the obtained H_2_O_2_ to the amount of contained TiO_2_ catalyst weight as shown in Figure S19). The enhanced productivity in continuous flow is attributed to an improved mass transfer due to the generated slug flow and the increased surface-to-volume ratio of the catalyst suspension. This consequently leads to a higher availability of oxygen as the reactant for the photocatalytic reduction reaction.

In the case of a maintained amount of photocatalyst components (Figure 6c and d), the overall amount of supraparticles per reaction increases with higher SiO_2_ contents. In the batch reactions, the produced H_2_O_2_ concentration decreases with higher SiO_2_ contents in the case of supraparticles with a TiO_2_–SiO_2_ ratio of 1:1 up to 1:6. This is attributed to enhanced scattering of the UV light due to a high supraparticle concentration in the reaction. The overall light interaction is thus reduced, while the contained catalytically active component in a single supraparticle should still be able to easily interact with light due to the supraparticle structure. The same effect can be observed in continuous flow (reduction in the H_2_O_2_ concentration by roughly 50%).

## 4 Conclusion

Supraparticles consisting of either pure TiO_2_ or TiO_2_ and SiO_2_ nanoparticles in varying weight ratios were assembled by using the spray-drying process. H_2_O_2_ is generated by them via an oxygen reduction reaction, and its production reaches a steady state after a certain UV light irradiation time, which is typical for anatase-based photocatalysts. The synthesized supraparticles show significantly higher productivity compared to commercial P25 and P90. This is attributed to organic surface molecules from the wet-chemical TiO_2_ nanoparticle synthesis that prevent photodegradation of H_2_O_2_ to a considerable extent. Studying two different arrangements of the TiO_2_ and SiO_2_ nanoparticles within the supraparticle, namely a core–shell and an intermixed structure, it became evident that core–shell supraparticles produce significantly higher H_2_O_2_ amounts. This is caused by the higher number of TiO_2_ nanoparticles located at the outside of a supraparticle, which can directly interact with the UV light that approaches the supraparticle. An increase in SiO_2_ nanoparticle content within the TiO_2_–SiO_2_ core–shell supraparticles results in their enhanced mechanical stability, while their overall catalytic activity declines. Increasing concentrations of supraparticles result both in batch and in flow reactors in enhanced light scattering and thus a decrease in overall productivity, while the catalysts’ productivity is generally higher in flow reactions due to improved mass transfer and higher oxygen availability. The *in situ* synthesized H_2_O_2_ of the developed supraparticles can potentially be provided for cascade or downstream reactions, paving the way for efficient and sustainable syntheses by a controlled, safe, and needs-oriented production of H_2_O_2_.^52,53^ Additionally, this study displays a first step toward the future design of supraparticle-based hybrid catalysts for photoassisted cascade reactions.

## Supporting Information Available

Nanoparticle characterizations including materials and methods description, transmission as well as scanning electron microscopy, dynamic light scattering, X-ray diffraction, and zeta potential measurements; supraparticle characterizations including scanning electron microscopy, laser diffraction, nitrogen sorption, and measured hydrogen concentrations; photocatalysis setup photographs and calibration curve for the Eisenberg method (PDF)

## Abbreviations

BET: Brunauer–Emmett–Teller
FEP: fluorinated ethylene propylene
H_2_O_2_: hydrogen peroxide
NLDFT: nonlocal density functional theory
PSD: pore size distribution
SiO_2_: silica
TiO_2_: titania

## References

[ref1] van Oers M. C. M.; Rutjes F. P. J. T.; van Hest J. C. M. Cascade Reactions in Nanoreactors. Curr. Opin. Biotechnol. 2014, 28, 10–16. 10.1016/j.copbio.2013.10.011. 24832069

[ref2] Yang N.; Tian Y.; Zhang M.; Peng X.; Li F.; Li J.; Li Y.; Fan B.; Wang F.; Song H. Photocatalyst-Enzyme Hybrid Systems for Light-Driven Biotransformation. Biotechnology advances 2022, 54, 107808 10.1016/j.biotechadv.2021.107808. 34324993

[ref3] Poe S. L.; Kobaslija M.; McQuade D. T. Mechanism and Application of a Microcapsule Enabled Multicatalyst Reaction. J. Am. Chem. Soc. 2007, 129, 9216–9221. 10.1021/ja071706x. 17602626

[ref4] Wapshott-Stehli H. L.; Grunden A. M. In Situ H_2_O_2_ Generation Methods in the Context of Enzyme Biocatalysis. Enzyme Microb. Technol. 2021, 145, 109744 10.1016/j.enzmictec.2021.109744. 33750536

[ref5] Gao S.; Lin H.; Zhang H.; Yao H.; Chen Y.; Shi J. Nanocatalytic Tumor Therapy by Biomimetic Dual Inorganic Nanozyme-Catalyzed Cascade Reaction. Advanced science (Weinheim, Baden-Wurttemberg, Germany) 2019, 6, 1801733 10.1002/advs.201801733. 31168441 PMC6364502

[ref6] Lewis R. J.; Hutchings G. J. Selective Oxidation Using In Situ-Generated Hydrogen Peroxide. Accounts of chemical research 2024, 57, 106–119. 10.1021/acs.accounts.3c00581. 38116936 PMC10765371

[ref7] García-Serna J.; Moreno T.; Biasi P.; Cocero M. J.; Mikkola J.-P.; Salmi T. O. Engineering in Direct Synthesis of Hydrogen Peroxide: Targets, Reactors and Guidelines for Operational Conditions. Green Chem. 2014, 16, 2320 10.1039/c3gc41600c.

[ref8] Edwards J. K.; Freakley S. J.; Lewis R. J.; Pritchard J. C.; Hutchings G. J. Advances in the Direct Synthesis of Hydrogen Peroxide from Hydrogen and Oxygen. Catal. Today 2015, 248, 3–9. 10.1016/j.cattod.2014.03.011.

[ref9] Pangotra D.; Csepei L.-I.; Roth A.; Sieber V.; Vieira L. Anodic Generation of Hydrogen Peroxide in Continuous Flow. Green Chem. 2022, 24, 7931–7940. 10.1039/D2GC02575B.

[ref10] Giraudeau P.; Felpin F.-X. Flow Reactors Integrated with in-Line Monitoring Using Benchtop NMR Spectroscopy. React. Chem. Eng. 2018, 3, 399–413. 10.1039/C8RE00083B.

[ref11] Browne D. L.; Wright S.; Deadman B. J.; Dunnage S.; Baxendale I. R.; Turner R. M.; Ley S. V. Continuous Flow Reaction Monitoring Using an on-Line Miniature Mass Spectrometer. Rapid communications in mass spectrometry: RCM 2012, 26, 1999–2010. 10.1002/rcm.6312. 22847699

[ref12] Fabry D. C.; Sugiono E.; Rueping M. Online Monitoring and Analysis for Autonomous Continuous Flow Self-Optimizing Reactor Systems. React. Chem. Eng. 2016, 1, 129–133. 10.1039/C5RE00038F.

[ref13] Perro A.; Lebourdon G.; Henry S.; Lecomte S.; Servant L.; Marre S. Combining Microfluidics and FT-IR Spectroscopy: towards Spatially Resolved Information on Chemical Processes. React. Chem. Eng. 2016, 1, 577–594. 10.1039/C6RE00127K.

[ref14] Silva Elipe M. V.; Milburn R. R. Monitoring Chemical Reactions by Low-Field Benchtop NMR at 45 MHz: Pros and Cons. Magnetic resonance in chemistry: MRC 2016, 54, 437–443. 10.1002/mrc.4189. 25616193

[ref15] Gutmann B.; Cantillo D.; Kappe C. O. Continuous-Flow Technology—a Tool for the Safe Manufacturing of Active Pharmaceutical Ingredients. Angewandte Chemie (International ed. in English) 2015, 54, 6688–6728. 10.1002/anie.201409318. 25989203

[ref16] Sambiagio C.; Noël T. Flow Photochemistry: Shine Some Light on Those Tubes!. TRECHEM 2020, 2, 92–106. 10.1016/j.trechm.2019.09.003.

[ref17] Constantino D. S.; Dias M. M.; Silva A. M.; Faria J. L.; Silva C. G. Intensification Strategies for Improving the Performance of Photocatalytic Processes: A review. Journal of Cleaner Production 2022, 340, 130800 10.1016/j.jclepro.2022.130800.

[ref18] Wintzheimer S.; Szczerba W.; GuilhermeBuzanich A.; Kashiwaya S.; Klein A.; Jaegermann W.; Toupance T.; Shmeliov A.; Nicolosi V.; Heuzé K.; Mandel K.; Dembski S. Discovering the Determining Parameters for the Photocatalytic Activity of TiO_2_ Colloids Based on an Anomalous Dependence on the Specific Surface Area. Part & Part Syst. Charact 2018, 35, 1800216 10.1002/ppsc.201800216.

[ref19] Wintzheimer S.; Luthardt L.; Le Cao K. A.; Imaz I.; Maspoch D.; Ogi T.; Bück A.; Debecker D. P.; Faustini M.; Mandel K. Multifunctional, Hybrid Materials Design via Spray-Drying: Much more than Just Drying. Advanced materials (Deerfield Beach, Fla.) 2023, 35, e2306648 10.1002/adma.202306648. 37840431

[ref20] Biskupski D.; Herbig B.; Schottner G.; Moos R. Nanosized Titania Derived from a Novel Sol–Gel Process for Ammonia Gas Sensor Applications. Sens. Actuators, B 2011, 153, 329–334. 10.1016/j.snb.2010.10.029.

[ref21] Herbig B.; Löbmann P. Mesoporous TiO_2_ Thin Films Prepared from Hydrothermally Treated Precursor Powder Sols. J. Sol-Gel Sci. Technol. 2018, 87, 292–298. 10.1007/s10971-018-4730-y.

[ref22] Wenderoth S.; Granath T.; Prieschl J.; Wintzheimer S.; Mandel K. Abrasion Indicators for Smart Surfaces Based on a Luminescence Turn-On Effect in Supraparticles. Advanced Photonics Research 2020, 1, 2000023 10.1002/adpr.202000023.

[ref23] Chang Q.; Tang H. Immobilization of Horseradish Peroxidase on NH_2_-Modified Magnetic Fe_3_O_4_/SiO_2_ Particles and its Application in Removal of 2,4-Dichlorophenol. Molecules (Basel, Switzerland) 2014, 19, 15768–15782. 10.3390/molecules191015768. 25268726 PMC6271698

[ref24] Eisenberg G. Colorimetric Determination of Hydrogen Peroxide. Ind. Eng. Chem. Anal. Ed. 1943, 15, 327–328. 10.1021/i560117a011.

[ref25] Wintzheimer S.; Miller F.; Prieschl J.; Retter M.; Mandel K. Supraparticles with Silica Protection for Redispersible, Calcined Nanoparticles. Nanoscale advances 2019, 1, 4277–4281. 10.1039/C9NA00442D. 36134422 PMC9417870

[ref26] Alesanco Y.; Palenzuela J.; Tena-Zaera R.; Cabañero G.; Grande H.; Herbig B.; Schmitt A.; Schott M.; Posset U.; Guerfi A.; Dontigny M.; Zaghib K.; Viñuales A. Plastic Electrochromic Devices Based on Viologen-Modified TiO_2_ Films Prepared at Low Temperature. Sol. Energy Mater. Sol. Cells 2016, 157, 624–635. 10.1016/j.solmat.2016.07.034.

[ref27] Viñuales A.; Herbig B.; Alesanco Y.; Palenzuela J.; Rodriguez J.; Schmitt A.; Posset U. One-Step Preparation of Viologen-TiO2 Nanoparticles via a Hydrothermally Assisted Sol–Gel Process for Use in Electrochromic Films and Devices. Part & Part Syst. Charact 2018, 35, 1800142 10.1002/ppsc.201800142.

[ref28] Reichstein J.; Schötz S.; Macht M.; Maisel S.; Stockinger N.; Collados C. C.; Schubert K.; Blaumeiser D.; Wintzheimer S.; Görling A.; Thommes M.; Zahn D.; Libuda J.; Bauer T.; Mandel K. Supraparticles for Bare-Eye H_2_ Indication and Monitoring: Design, Working Principle, and Molecular Mobility. Adv. Funct Materials 2022, 32, 2112379 10.1002/adfm.202112379.

[ref29] Li L.; Xu L.; Hu Z.; Yu J. C. Enhanced Mass Transfer of Oxygen through a Gas–Liquid–Solid Interface for Photocatalytic Hydrogen Peroxide Production. Adv. Funct Materials 2021, 31, 2106120 10.1002/adfm.202106120.

[ref30] Hou H.; Zeng X.; Zhang X. Production of Hydrogen Peroxide by Photocatalytic Processes. Angewandte Chemie (International ed. in English) 2020, 59, 17356–17376. 10.1002/anie.201911609. 31571331

[ref31] Burek B. O.; Bahnemann D. W.; Bloh J. Z. Modeling and Optimization of the Photocatalytic Reduction of Molecular Oxygen to Hydrogen Peroxide over Titanium Dioxide. ACS Catal. 2019, 9, 25–37. 10.1021/acscatal.8b03638.

[ref32] Kormann C.; Bahnemann D. W.; Hoffmann M. R. Photocatalytic Production of Hydrogen Peroxides and Organic Peroxides in Aqueous Suspensions of Titanium Dioxide, Zinc Oxide, and Desert Sand. Environ. Sci. Technol. 1988, 22, 798–806. 10.1021/es00172a009. 22195664

[ref33] Li X.; Chen C.; Zhao J. Mechanism of Photodecomposition of H_2_O_2_ on TiO_2_ Surfaces under Visible Light Irradiation. Langmuir 2001, 17, 4118–4122. 10.1021/la010035s.

[ref34] Yi J.; Bahrini C.; Schoemaecker C.; Fittschen C.; Choi W. Photocatalytic Decomposition of H_2_O_2_ on Different TiO2 Surfaces Along with the Concurrent Generation of HO_2_ Radicals Monitored Using Cavity Ring Down Spectroscopy. J. Phys. Chem. C 2012, 116, 10090–10097. 10.1021/jp301405e.

[ref35] Lousada C. M.; Johansson A. J.; Brinck T.; Jonsson M. Mechanism of H_2_O_2_ Decomposition on Transition Metal Oxide Surfaces. J. Phys. Chem. C 2012, 116, 9533–9543. 10.1021/jp300255h.

[ref36] FANG W.; WANG L.; LI C. Preparation of Au-OVs-BiOBr-P25 Z-Scheme Photocatalyst and its Photocatalytic Performance in Overall Water Splitting. Journal of Fuel Chemistry and Technology 2022, 50, 446–454. 10.1016/S1872-5813(21)60174-3.

[ref37] Salehi Ghalehsefid E.; Ghorbani Jahani Z.; Aliabadi A.; Ghodrati M.; Khamesan A.; Parsaei-Khomami A.; Mousavi M.; Hosseini M.-A.; Ghasemi J. B.; Li X. TiO_2_ Nanotube/ZnIn_2_S_4_ Nanoflower Composite with Step-Scheme Heterojunction for Efficient Photocatalytic H_2_O_2_ Production and Organic Dye Degradation. Journal of Environmental Chemical Engineering 2023, 11, 110160 10.1016/j.jece.2023.110160.

[ref38] Sahel K.; Elsellami L.; Mirali I.; Dappozze F.; Bouhent M.; Guillard C. Hydrogen Peroxide and Photocatalysis. Applied Catalysis B: Environmental 2016, 188, 106–112. 10.1016/j.apcatb.2015.12.044.

[ref39] Gößl D.; Singer H.; Chiu H.-Y.; Schmidt A.; Lichtnecker M.; Engelke H.; Bein T. Highly Active Enzymes Immobilized in Large Pore Colloidal Mesoporous Silica Nanoparticles. New J. Chem. 2019, 43, 1671–1680. 10.1039/C8NJ04585B.

[ref40] Kim J.; Jia H.; Lee C.; Chung S.; Kwak J. H.; Shin Y.; Dohnalkova A.; Kim B.-G.; Wang P.; Grate J. W. Single Enzyme Nanoparticles in Nanoporous Silica: A Hierarchical Approach to Enzyme Stabilization and Immobilization. Enzyme Microb. Technol. 2006, 39, 474–480. 10.1016/j.enzmictec.2005.11.042.

[ref41] Popat A.; Hartono S. B.; Stahr F.; Liu J.; Qiao S. Z.; Qing Max Lu G. Mesoporous Silica Nanoparticles for Bioadsorption, Enzyme Immobilisation, and Delivery Carriers. Nanoscale 2011, 3, 2801–2818. 10.1039/c1nr10224a. 21547299

[ref42] Qhobosheane M.; Santra S.; Zhang P.; Tan W. Biochemically Functionalized Silica Nanoparticles. Analyst 2001, 126, 1274–1278. 10.1039/b101489g. 11534592

[ref43] Lee S. H.; Ryu J.; Nam D. H.; Park C. B. Photoenzymatic Synthesis through Sustainable NADH Regeneration by SiO2-Supported Quantum Dots. Chemical communications (Cambridge, England) 2011, 47, 4643–4645. 10.1039/c0cc05246a. 21336344

[ref44] Zhou H.; Groppe P.; Zimmermann T.; Wintzheimer S.; Mandel K. Influence of Cation Concentration and Valence on the Structure and Texture of Spray-Dried Supraparticles from Colloidal Silica Dispersions. J. Colloid Interface Sci. 2024, 658, 199–208. 10.1016/j.jcis.2023.12.051. 38100976

[ref45] Arutanti O.; Nandiyanto A. B. D.; Ogi T.; Kim T. O.; Okuyama K. Influences of Porous Structurization and Pt Addition on the Improvement of Photocatalytic Performance of WO_3_ particles. ACS Appl. Mater. Interfaces 2015, 7, 3009–3017. 10.1021/am507935j. 25608579

[ref46] Migneault I.; Dartiguenave C.; Bertrand M. J.; Waldron K. C. Glutaraldehyde: Behavior in Aqueous Solution, Reaction with Proteins, and Application to Enzyme Crosslinking. BioTechniques 2004, 37, 790–802. 10.2144/04375RV01. 15560135

[ref47] Stauch C.; Ballweg T.; Stracke W.; Luxenhofer R.; Mandel K. Burstable Nanostructured Micro-Raspberries: Towards Redispersible Nanoparticles from Dry Powders. J. Colloid Interface Sci. 2017, 490, 401–409. 10.1016/j.jcis.2016.11.047. 27914339

[ref48] Khan H.; Rigamonti M. G.; Patience G. S.; Boffito D. C. Spray Dried TiO_2_/WO_3_ Heterostructure for Photocatalytic Applications with Residual Activity in the Dark. Applied Catalysis B: Environmental 2018, 226, 311–323. 10.1016/j.apcatb.2017.12.049.

[ref49] Khan H.; Usen N.; Boffito D. C. Spray-Dried Microporous Pt/TiO_2_ Degrades 4-Chlorophenol under UV and Visible Light. Journal of Environmental Chemical Engineering 2019, 7, 103267 10.1016/j.jece.2019.103267.

[ref50] Le P. H.; Kitamoto Y.; Yamashita S.; Le Cao K. A.; Hirano T.; Amen T. W. M.; Tsunoji N.; Ogi T. Macropore-Size Engineering toward Enhancing the Catalytic Performance of CO Oxidation over Three-Way Catalyst Particles. ACS Appl. Mater. Interfaces 2023, 15, 54073–54084. 10.1021/acsami.3c11489. 37944066

[ref51] Pieber B.; Shalom M.; Antonietti M.; Seeberger P. H.; Gilmore K. Continuous Heterogeneous Photocatalysis in Serial Micro-Batch Reactors. Angewandte Chemie (International ed. in English) 2018, 57, 9976–9979. 10.1002/anie.201712568. 29377383

[ref52] Zhang W.; Fueyo E. F.; Hollmann F.; Martin L. L.; Pesic M.; Wardenga R.; Höhne M.; Schmidt S. Combining Photo-Organo Redox- and Enzyme Catalysis Facilitates Asymmetric C-H Bond Functionalization. Eur. J. Org. Chem. 2019, 2019, 80–84. 10.1002/ejoc.201801692. PMC6470836 31007570

[ref53] Schmermund L.; Reischauer S.; Bierbaumer S.; Winkler C. K.; Diaz-Rodriguez A.; Edwards L. J.; Kara S.; Mielke T.; Cartwright J.; Grogan G.; Pieber B.; Kroutil W. Chromoselective Photocatalysis Enables Stereocomplementary Biocatalytic Pathways*. Angewandte Chemie (International ed. in English) 2021, 60, 6965–6969. 10.1002/anie.202100164. 33529432 PMC8048449

