# Supplementary material for: Hydrogen Peroxide Producing Titania-Silica Supraparticles as Tailorable Photocatalysts for Flow Chemistry Reactions in Microfluidic Reactors
Source: Chem Bio Eng. 2025 Jan 16;2(3):199–209. doi: 10.1021/cbe.4c00154 (PMC11955856; doi:10.1021/cbe.4c00154)
Supplement: Supplementary file 1 — be4c00154_si_001.pdf [file be4c00154_si_001.pdf]

Supporting Information

Hydrogen peroxide producing titania-silica supraparticles as tailorable photocatalysts for flow chemistry reactions in microfluidic reactors

Bettina Herbig,[*a] Egzon Cermjani,[b,c] Doris Hanselmann,[a] Angelika Schmitt,[a] Christoph Deckers,[b] Thomas H. Rehm,[b] Karl Mandel,[a,d] Susanne Wintzheimer[a,d]*

a Fraunhofer-Institute for Silicate Research ISC, Neunerplatz 2, D97082 Würzburg, Germany.

b Fraunhofer-Institut für Mikrotechnik und Mikrosysteme IMM, Carl-Zeiss-Str. 18-20, D55129 Mainz, Germany.

c Johannes Gutenberg-University Mainz, Department of Chemistry, Duesbergweg 10-14, 55128 Mainz, Germany.

d Department of Chemistry and Pharmacy, Inorganic Chemistry, Friedrich-Alexander-University Erlangen-Nürnberg, Egerlandstrasse 1, D91058 Erlangen, Germany.

Corresponding author: bettina.herbig@isc.fraunhofer.de

Materials and Methods – Nanoparticle characterizations

The hydrodynamic sizes (intensity-weighted) and zeta-potentials of the titania and silica nanoparticles were determined using dynamic light scattering (DLS, Zetasizer Nano ZS from Malvern Instruments in combination with the Multi-Purpose Titrator MPT-2). The isoelectric points of the nanoparticles were determined by titrating with HCl and NaOH. Transmission electron microscopy (TEM) with a HITACHI H7650 at an acceleration voltage of 100 kV was used to examine the nanoparticle morphology (with samples prepared on carbon-coated copper grids). The as synthesized TiO_2 nanoparticle sample was analyzed via XRD on a PANalytical Empyrean Series 2 employing Cu Kα radiation.

The spray-dried titania-silica supraparticles were characterized using scanning electron microscopy (SEM, Zeiss Supra 25, Germany) coupled with an EDAX Element EDS System for energy dispersive x-ray spectroscopy. The working distance was 8.5 mm and the acceleration voltage for EDS was 10 kV. Nitrogen sorption measurements were conducted to determine the pore sizes and pore volumes of the obtained supraparticles. The nitrogen isotherms were performed on a NOVAtouch LX2 (AntonPaar) at 77 K. Before measurements, the samples were dried at 30 mbar at 115 °C for 16 h in a vacuum drying chamber (VO29, Mammert) and degassed at 350 °C for 12 h under vacuum.

Results and Discussion

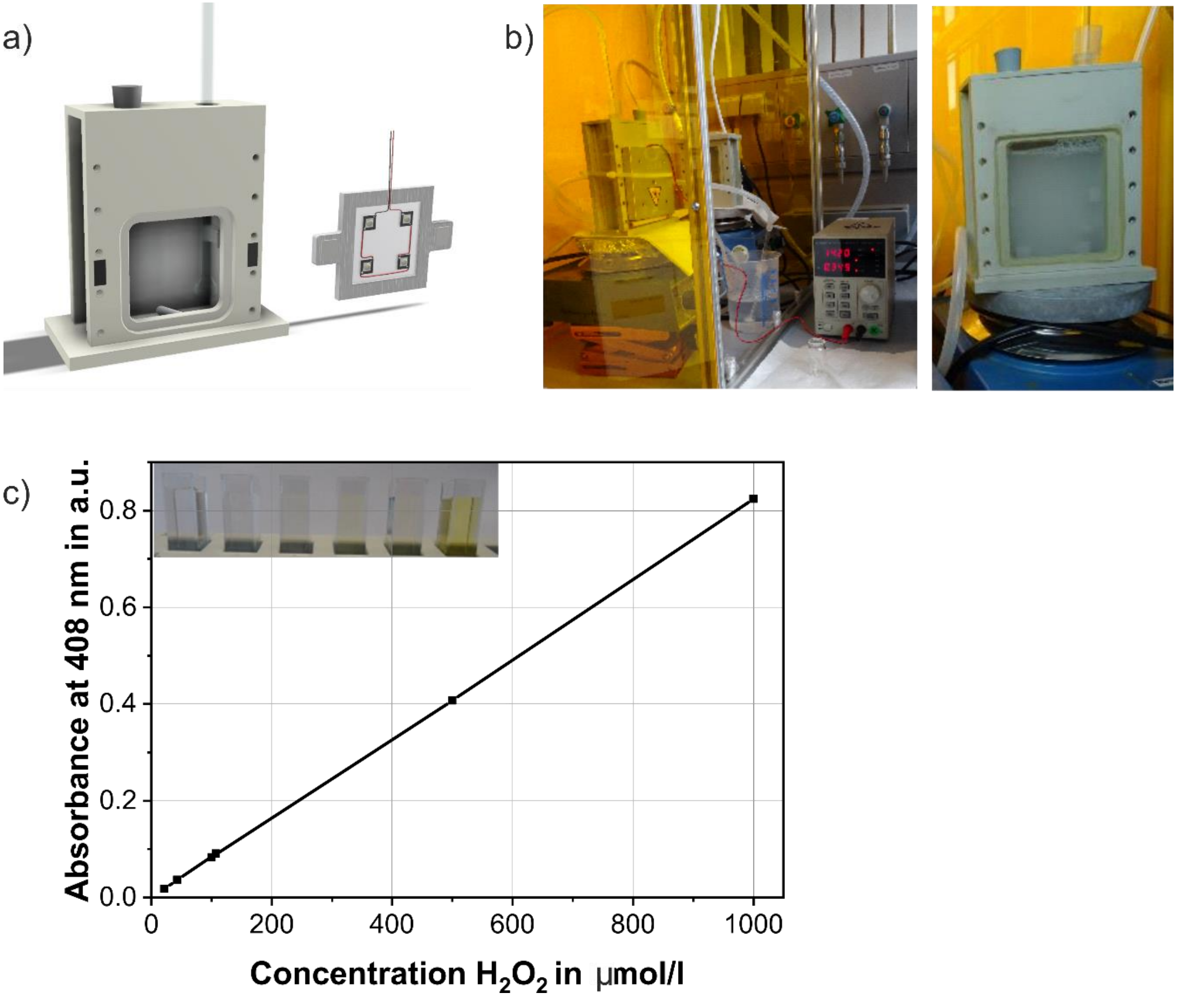

Figure S1. Photocatalysis setup as a) CAD drawing and b) photographs showing the reaction chamber, the compressed air inlet and the LED. c) Calibration curve for the determination of the concentration of generated H_2O_2 using the Eisenberg method.

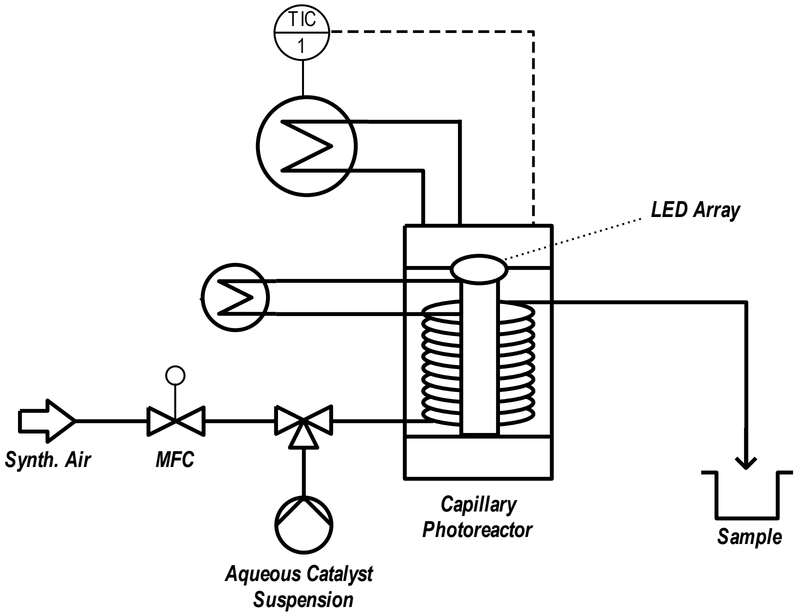

Figure S2. Setup for the continuous flow production of H_2O_2. MFC = Mass Flow Controller. TIC = Temperature Indicator Control. $V_{\text{Capillary Photoreactor}}$ = 30 mL. T = 25 °C.

Table S1. Hardware components installed for continuous flow reactions.

Hardware component	Specifications/Model	Supplier
Syringe pump	Model: Fusion 4000 X	Chemyx
Cryostat for LED-array and capillary reactor	F10 with HC E07 – control unit	Julabo GmbH
Tubing	1/8” FEP-capillary	Bolender GmbH
Power Supply for LED-array	Voltcraft LSP-1403	Conrad Electronic SE
LEDs	UV-A (365 nm), 6x6 Nichia NCSU276AT	Nichia Corporation, Japan & Avonec, Germany

Table S2. Electronic settings for the 6x6 LED-array (365 nm).

**Voltage [V]**	**Current [A]**	**El. Power of array $P_{el.}$ [$W_{el.}$]**	**Radiant flux [W]**
23.3	2.1	50	19.6

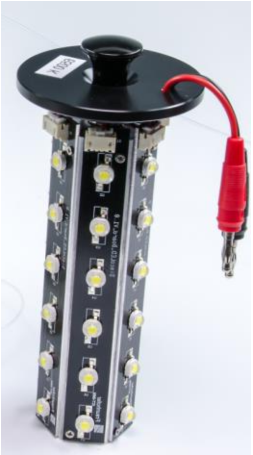

Figure S3. Hexagonal 6x6 LED-Array for insertion in the capillary photoreactor.

Table S3. Reaction parameters for continuous flow synthesis of hydrogen peroxide with different residence times/UV-light exposure durations. λ = 365 nm. T = 25 °C. $V_{\text{Suspension}}$ = 20 mL. V_{Reactor} = 30 mL.

Entry	Supraparticle	w% (Supraparticles)	Residence time τ [min]	Flow rate (synth. air) [mL/min]	Flow rate (suspension) [mL/min]	c (H_2O_2) [uM]
1	TiO_2	0.05	5	4.500	1.500	142.63
		0.05	10	2.250	0.750	164.84
		0.05	15	1.500	0.500	166.76
		0.05	20	1.125	0.375	145.14
2	TiO_2-SiO_2 (1:2)	0.05	5	4.500	1.500	51.26
		0.05	10	2.250	0.750	107.70
		0.05	15	1.500	0.500	93.30
		0.05	20	1.125	0.375	95.69

Table S4. Reaction parameters for continuous flow synthesis of hydrogen peroxide with different TiO_2-SiO_2 ratios and a constant amount of supraparticle. λ = 365 nm. T = 25 °C. $V_{Suspension}$ = 20 mL. $V_{Reactor}$ = 30 mL.

Entry	Supraparticle	w% (Supraparticle)	Residence time τ [min]	Flow rate (synth. air) [mL/min]	Flow rate (suspension) [mL/min]	c (H_2O_2) [uM]
1	TiO_2	0.05	5	4.5	1.5	142.63
2	TiO_2-SiO_2 (6:1)	0.05	5	4.5	1.5	106.89
3	TiO_2-SiO_2 (3:1)	0.05	5	4.5	1.5	93.27
4	TiO_2-SiO_2 (2:1)	0.05	5	4.5	1.5	94.11
5	TiO_2-SiO_2 (1:1)	0.05	5	4.5	1.5	87.22
6	TiO_2-SiO_2 (1:2)	0.05	5	4.5	1.5	81.90
7	TiO_2-SiO_2 (1:3)	0.05	5	4.5	1.5	45.69
8	TiO_2-SiO_2 (1:6)	0.05	5	4.5	1.5	25.69

Table S5. Reaction parameters for continuous flow synthesis of hydrogen peroxide with different TiO_2-SiO_2 ratios and a constant amount of TiO_2. λ = 365 nm. T = 25 °C. $V_{Suspension}$ = 20 mL. $V_{Reactor}$ = 30 mL.

Entry	Supraparticle	w% (Supraparticle)	Residence time τ [min]	Flow rate (synth. air) [mL/min]	Flow rate (suspension) [mL/min]	c (H_2O_2) [uM]
1	TiO_2	0.0500	5	4.5	1.5	142.63
2	TiO_2-SiO_2 (6:1)	0.0585	5	4.5	1.5	155.32
3	TiO_2-SiO_2 (3:1)	0.0665	5	4.5	1.5	157.75
4	TiO_2-SiO_2 (2:1)	0.0750	5	4.5	1.5	115.07
5	TiO_2-SiO_2 (1:1)	0.1000	5	4.5	1.5	124.37
6	TiO_2-SiO_2 (1:2)	0.1500	5	4.5	1.5	120.54
7	TiO_2-SiO_2 (1:3)	0.2000	5	4.5	1.5	91.19
8	TiO_2-SiO_2 (1:6)	0.3500	5	4.5	1.5	88.43

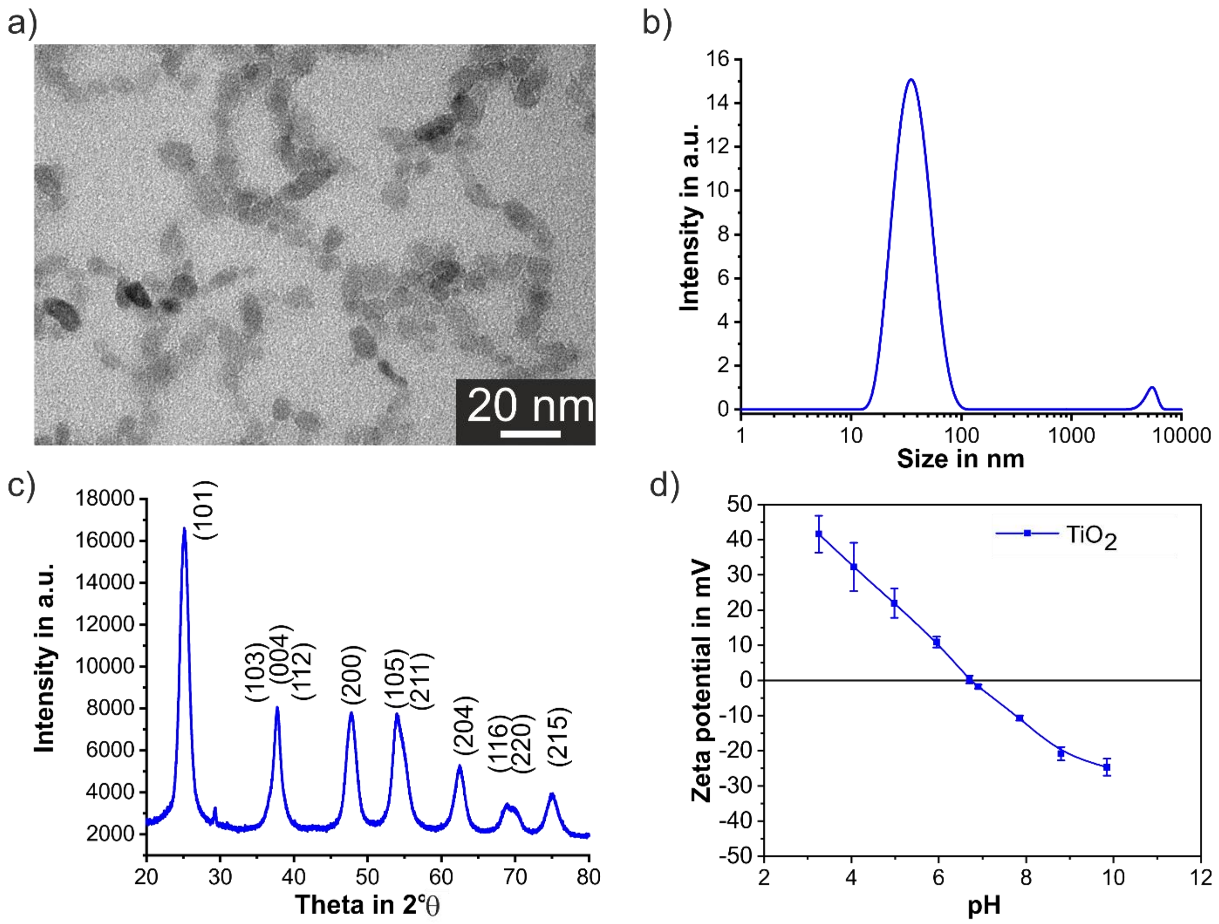

Figure S4. Characterizations of TiO_2 nanoparticles: a) transmission electron micrograph, b) dynamic light scattering hydrodynamic diameter distribution graph (intensity-weighted), c) X-ray diffraction pattern, and d) zeta potential measurements over the pH.

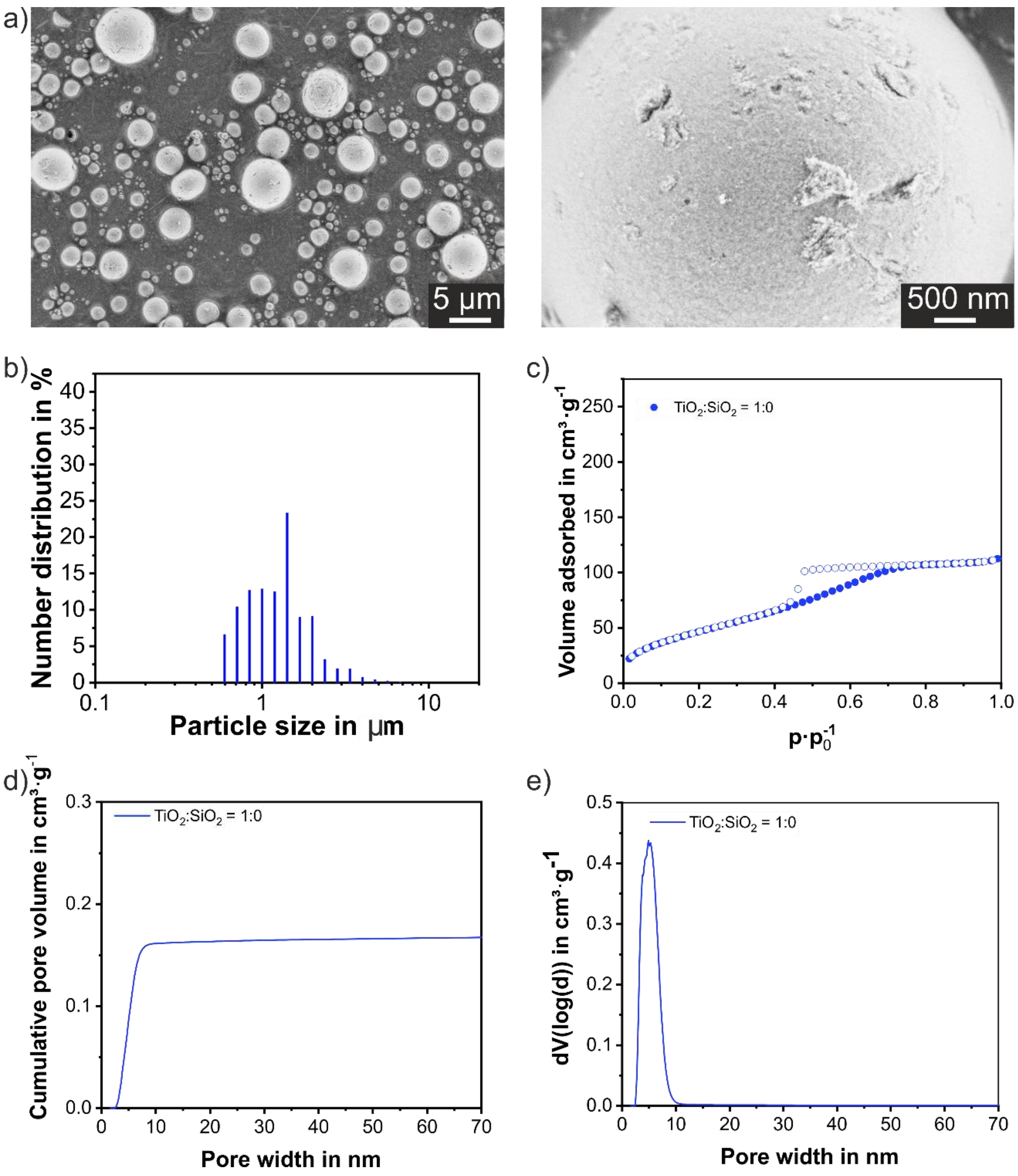

Figure S5. Characterizations of TiO_2 supraparticles: a) SEM overview micrograph showing the overall morphology and the size distribution of the spray-dried TiO_2 supraparticles as well as a close-up view of a single supraparticle surface, and b) number-weighted size distribution measured via laser diffraction. N_2 sorption analysis at 77 K shown as c) N_2 adsorption (full symbols) / desorption (open symbols) isotherms, d) cumulative pore volume obtained from NLDFT, as well as e) pore size distribution plot calculated by applying a dedicated NLDFT method on the adsorption branch of the isotherms assuming a cylindrical pore shape.

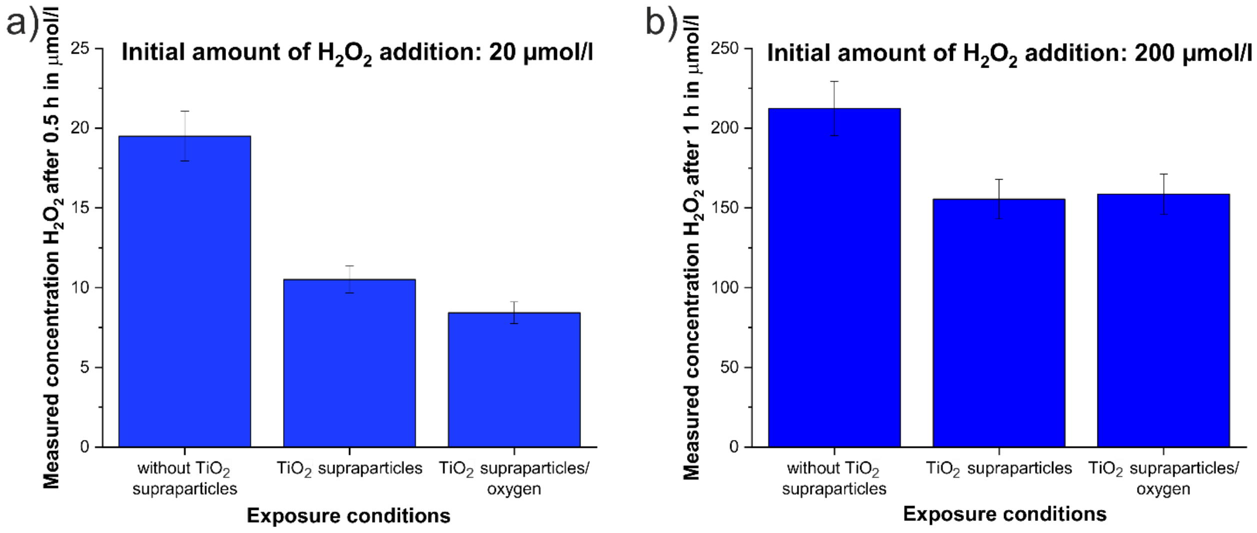

Figure S6. Still available amount of H_2O_2 in solution after a) 0.5 h and b) 1 h without UV light exposure and either without the presence of TiO_2 supraparticles, in presence of TiO_2 supraparticles, or in presence of TiO_2 supraparticles and additionally oxygen, indicating the adsorption of H_2O_2 on the supraparticle surface as pre-dominant mechanism of H_2O_2 removal in absence of UV light.

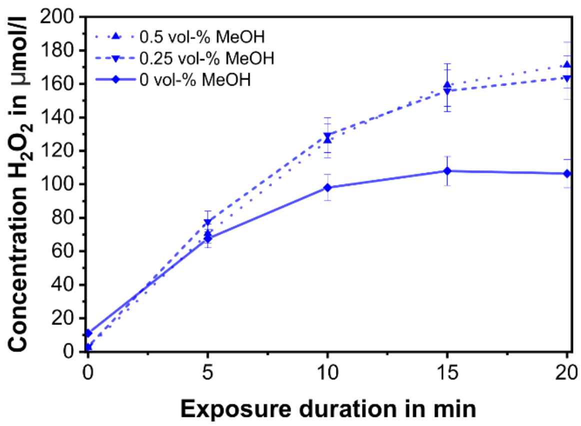

Figure S7. Photocatalytic H_2O_2 production over time upon addition of varying amounts of methanol as hole scavenger to the reaction batch (lines are only for guiding the eye).

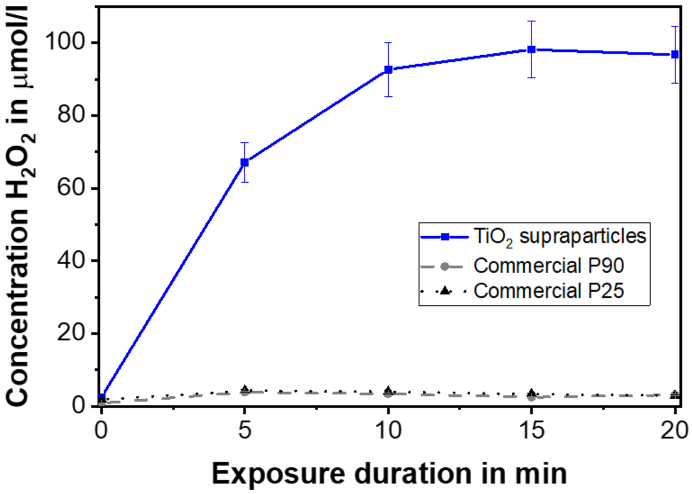

Figure S8. Photocatalytic H_2O_2 production over time using TiO_2 supraparticles, commercial P90 or commercial P25 TiO_2 particles as catalysts in the reaction (lines are only for guiding the eye).

Table S6. Comparison of the initial H_2O_2 production rate to relevant examples from the literature

Material	Available surface area [m^2 g^{-1}]	Irradiation wavelength [nm]	H_2O_2 determination method	H_2O_2 production rate within the first 5 mins of irradiation per mg catalyst [µmol l^{-1} min^{-1} mg^{-1}]	reference
TiO_2	n.a.	280 - 400 nm	redox titration with KMnO4	16	
TiO_2 P25	n.a.	360 nm	iodide oxidation	0.05	
Cu^+/TiO_2 P25	n.a.	300–400 nm	scopolectin dye oxidation	0.005	
TiO_2	8.1	λ > 300 nm	iodometric titration	0.2	
TiO_2, 2,3-dihydroxy-naphthalene and octa-decylphosphonic acid modified	285.5	λ > 450 nm	$[TiO(tpypH_4)]^{4+}$ complex titration method	0.67	
TiO_2 supraparticles	180	365 nm	Eisenberg method	0.104	This work

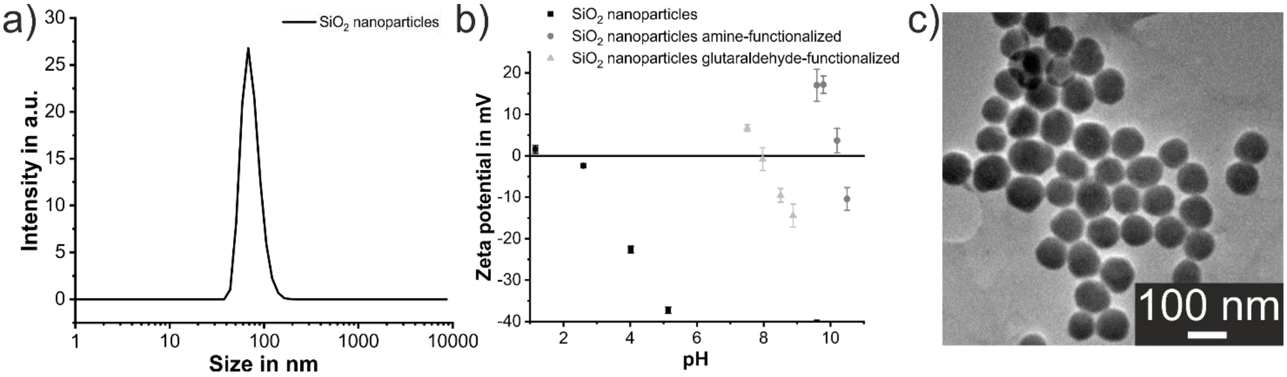

Figure S9. Characterizations of SiO_2 nanoparticles: a) dynamic light scattering hydrodynamic diameter distribution graph (intensity-weighted) of unmodified nanoparticles, b) zeta potential measurements over the pH of pure, aminopropyltriethoxysilane-functionalized, and subsequently glutaraldehyde-functionalized SiO_2 nanoparticles, c) transmission electron micrograph of unmodified nanoparticles.

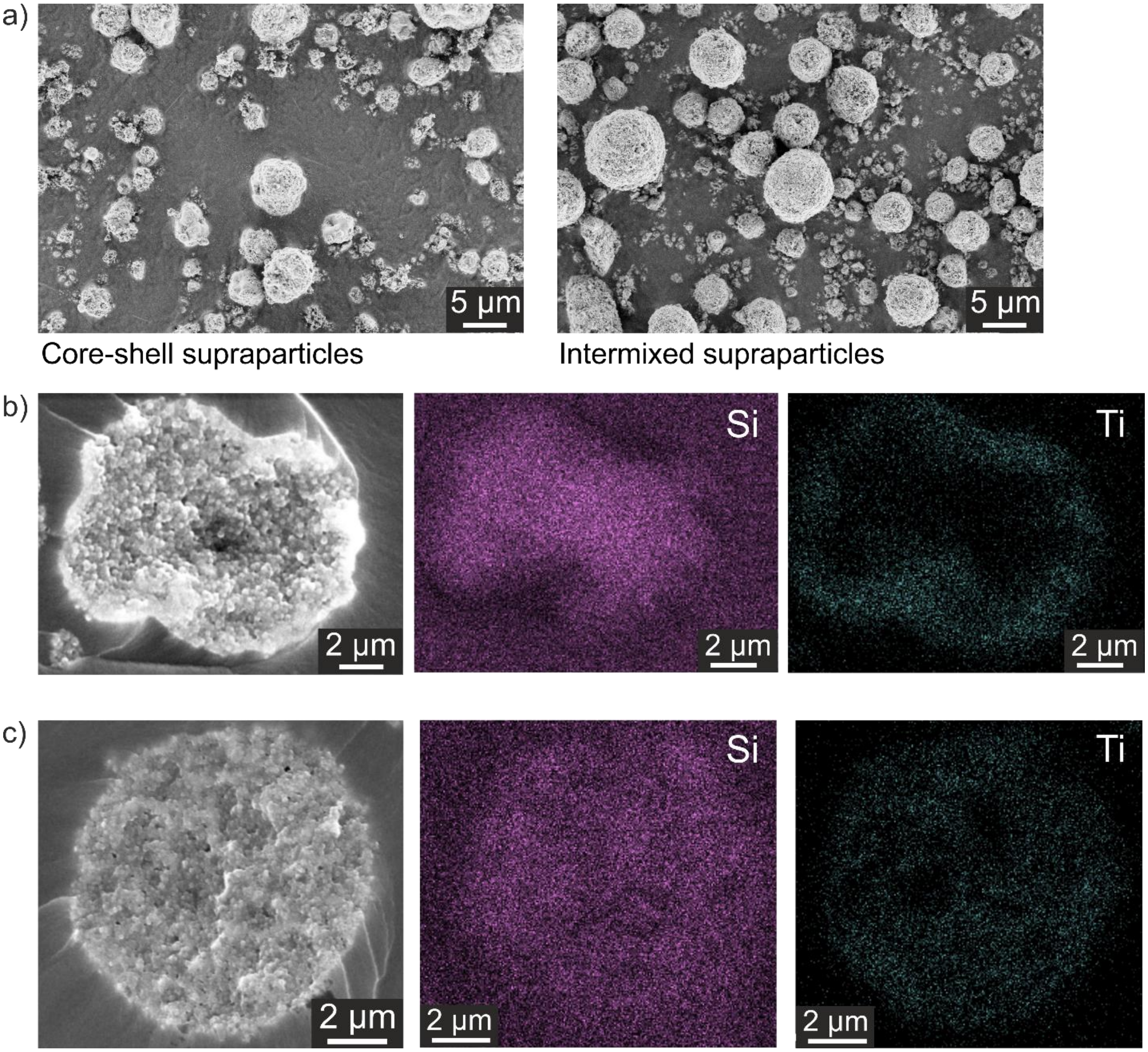

Figure S10. Characterizations of supraparticles consisting of TiO_2 and SiO_2 nanoparticles in a weight ratio of 1 to 2. The TiO_2-SiO_2 supraparticles were fabricated using either a stable or destabilized dispersion yielding core-shell or intermixed supraparticles respectively: a) SEM overview micrographs showing the overall morphology and the size distribution of the spray-dried TiO_2-SiO_2 supraparticles, as well as SEM-EDS of cross-sections of b) a core-shell and c) an intermixed supraparticle.

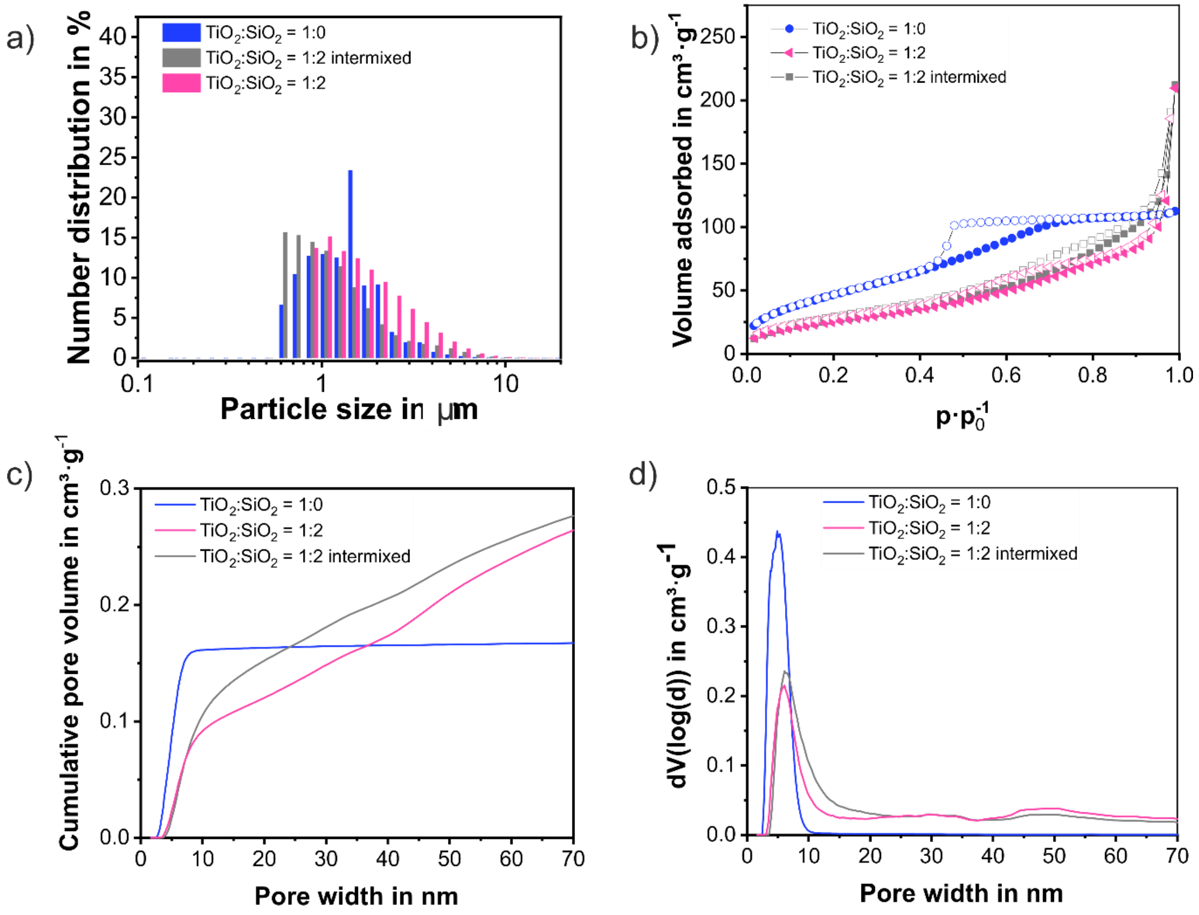

Figure S11. Characterizations of supraparticles consisting of TiO_2 and SiO_2 nanoparticles in a weight ratio of 1 to 2 in comparison to pure TiO_2 supraparticles. The TiO_2-SiO_2 supraparticles were fabricated using either a stable or destabilized dispersion yielding core-shell or intermixed supraparticles respectively: a) number-weighted size distribution measured via laser diffraction, N_2 sorption analysis at 77 K shown as b) N_2 adsorption (full symbols) / desorption (open symbols) isotherms, c) cumulative pore volume obtained from NLDFT, as well as d) pore size distribution plot calculated by applying a dedicated NLDFT method on the adsorption branch of the isotherms assuming a cylindrical pore shape.

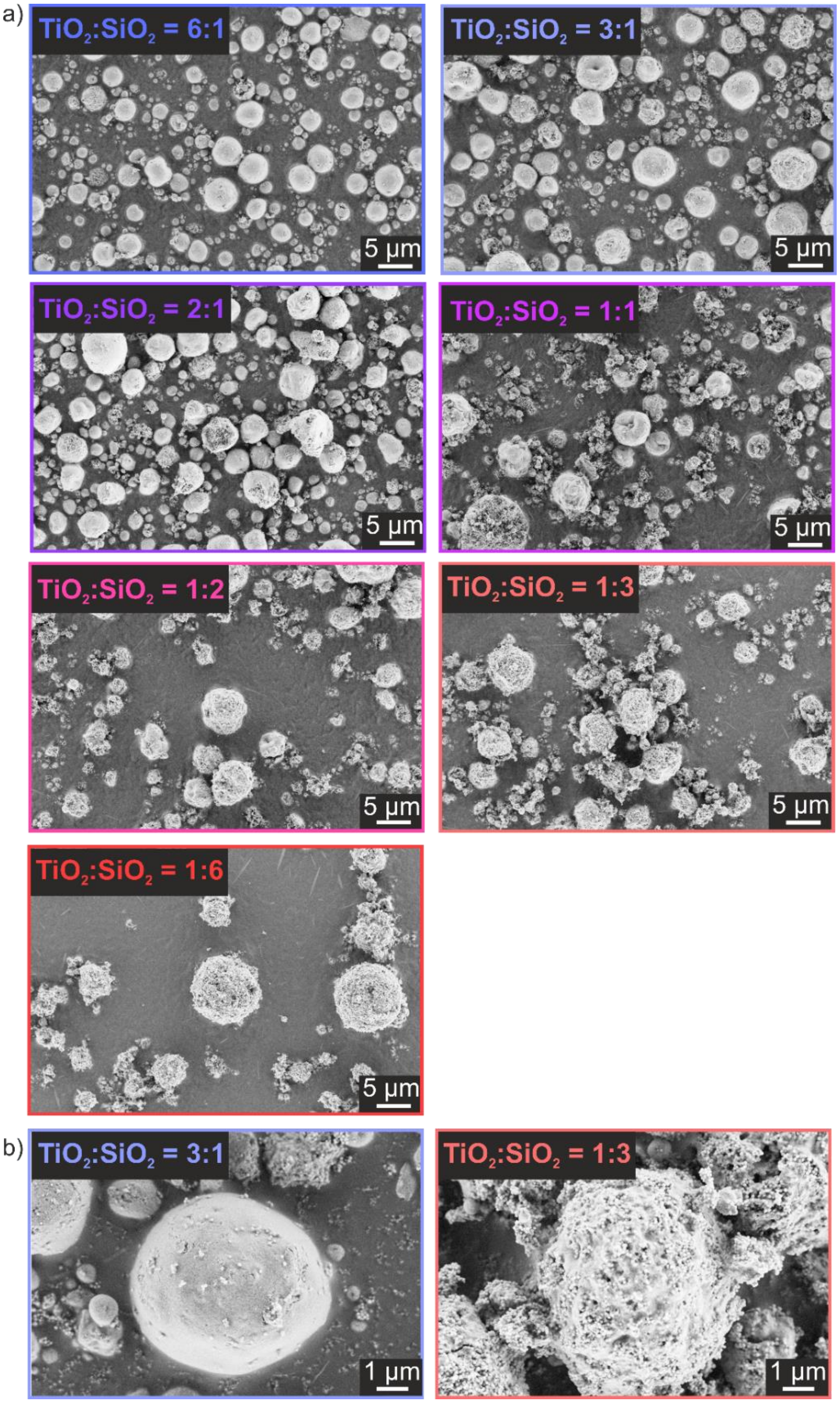

Figure S12. a) SEM overview micrographs showing the overall morphology and the size distribution of spray-dried supraparticles consisting of TiO_2 and SiO_2 nanoparticles in a weight ratio of 6:1, 3:1, 2:1, 1:1, 1:2, 1:3, and 1:6. b) Close-up views of spray-dried supraparticles consisting of TiO_2 and SiO_2 nanoparticles in a weight ratio of 3:1, and 1:3.

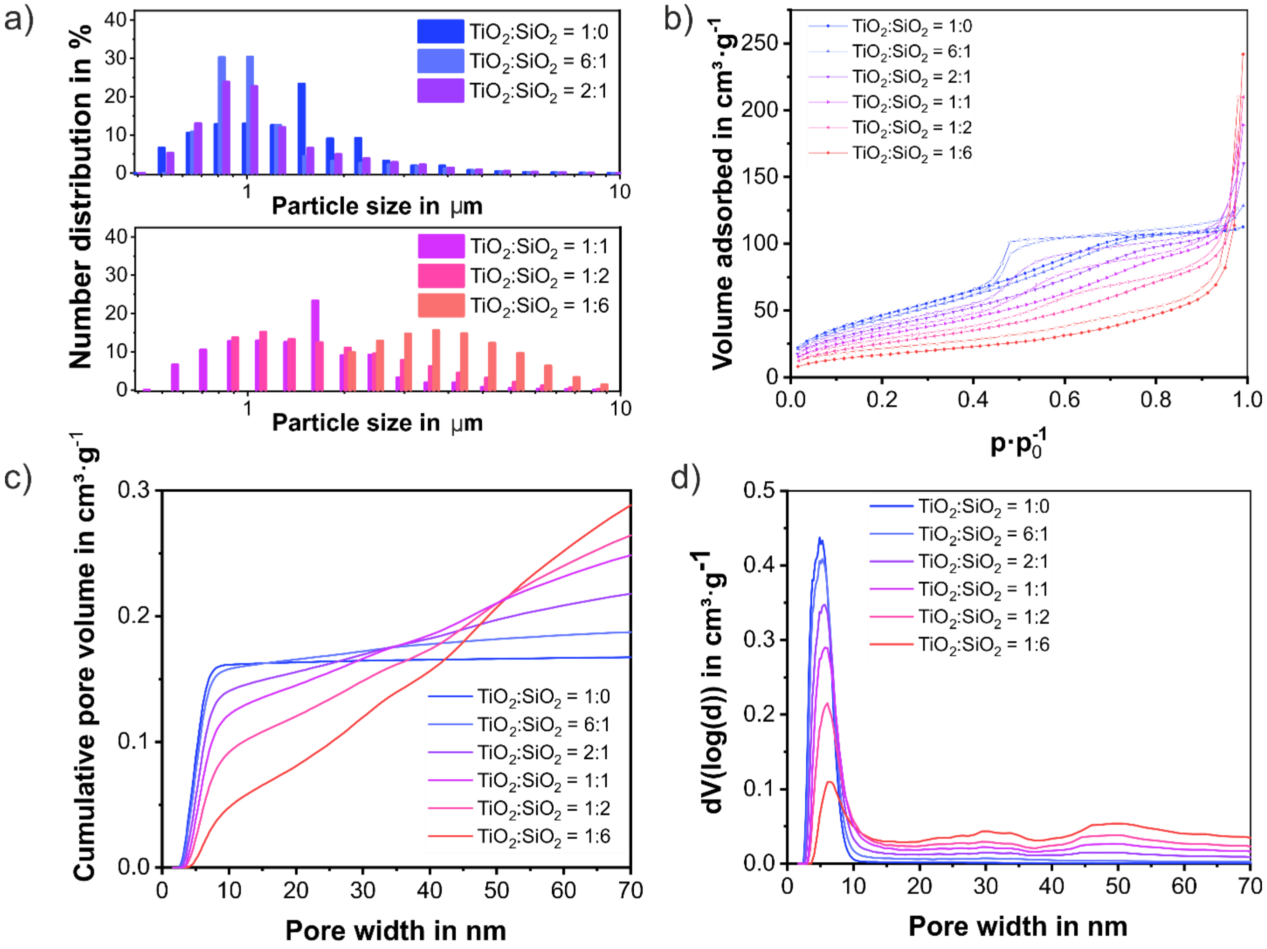

Figure S13. Characterizations of supraparticles consisting of TiO_2 and SiO_2 nanoparticles in a weight ratio of 6:1, 2:1, 1:1, 1:2, and 1:6 in comparison to pure TiO_2 supraparticles (1:0): a) number-weighted size distribution measured via laser diffraction, N_2 sorption analysis at 77 K shown as b) N_2 adsorption (full symbols) / desorption (open symbols) isotherms, c) cumulative pore volume obtained from NLDFT, and d) pore size distribution plot calculated by applying a dedicated NLDFT method on the adsorption branch of the isotherms assuming a cylindrical pore shape.

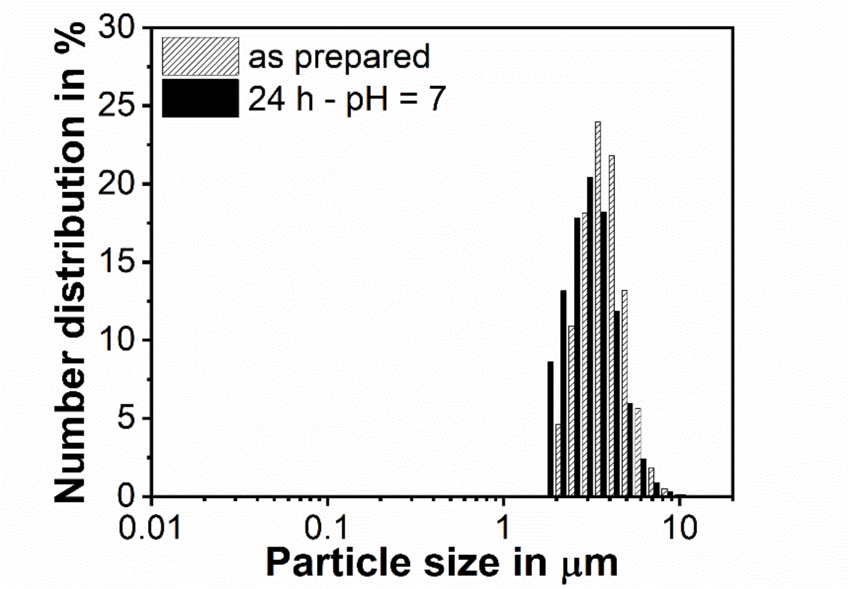

Figure S14. Number-weighted particle size distribution of glutaric acid-functionalized SiO_2 supraparticles as prepared and after stirring as dispersion in water for 24 h (pH = 7, using a magnetic stirring bar).

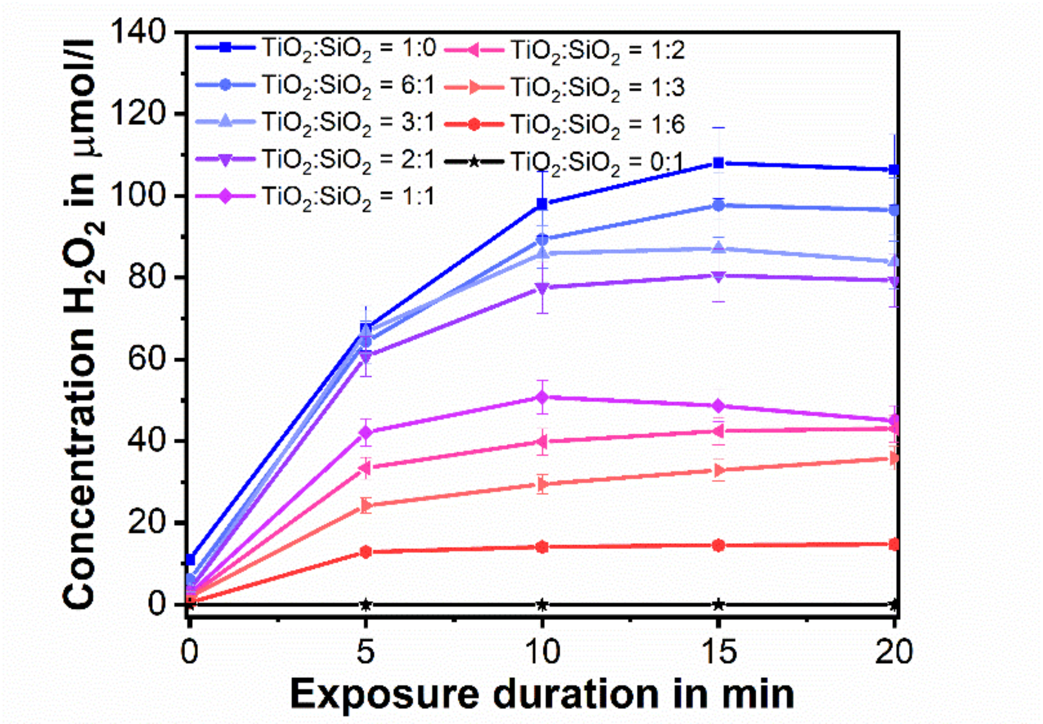

Figure S15. Cumulated concentration of H_2O_2 produced by supraparticles consisting of TiO_2, SiO_2 or TiO_2 and SiO_2 nanoparticles in a weight ratio of 6:1, 2:1, 1:1, 1:2, and 1:6 over the UV-light exposure duration (lines are only for guiding the eye).

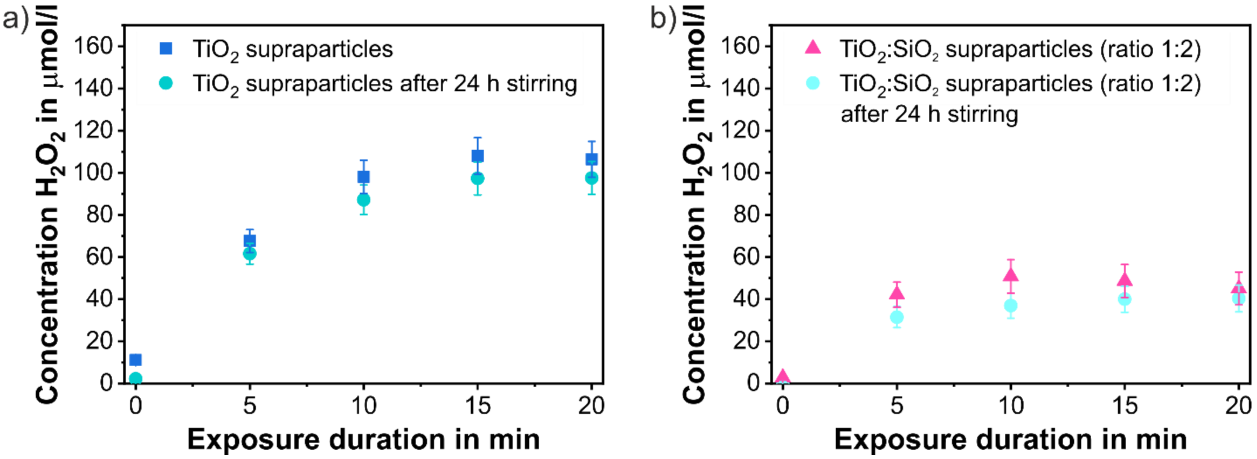

Figure S16. Cumulated concentration of H_2O_2 over the UV-light exposure duration produced by supraparticles consisting of TiO_2 nanoparticles (a) or TiO_2 and SiO_2 nanoparticles in a weight ratio of 1:2 (b) either as prepared or after stirring for 24 h.

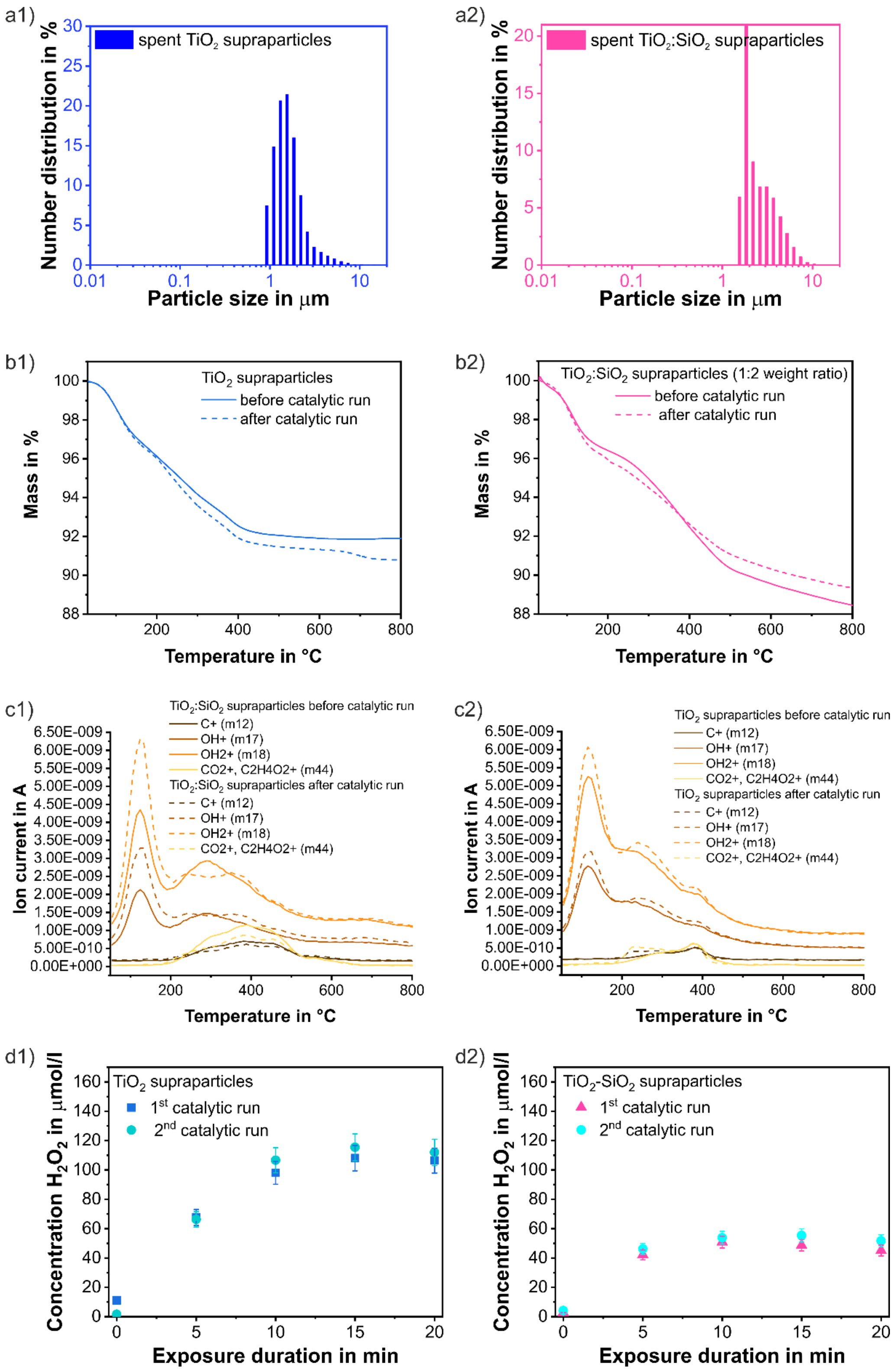

Figure S17. Characterizations of supraparticles consisting of TiO_2 nanoparticles (1) or TiO_2 and SiO_2 nanoparticles in a weight ratio of 1:2 (2): number-weighted size distribution measured via

laser diffraction of the spent catalysts after 1 catalytic run in batch mode (a), thermogravimetric analyses (TGA) of these catalysts at the start and after 1 catalytic run in batch mode (b), the corresponding evolved gas analysis by mass spectrometry during TGA of these catalysts at the start and after 1 catalytic run in batch mode (c), and the cumulated concentration of H_2O_2 over the UV-light exposure duration produced by these catalysts in the 1st and 2nd catalytic run in batch mode (d).

The diameter of the spent supraparticle catalysts does not significantly differ from the as-prepared samples and no smaller supraparticle fragments are detectable. The morphological supraparticle intactness is in agreement with SEM micrographs (Figure S18) of spent catalysts. Thermogravimetric analyses indicate the release of adsorbed water between 50 and 200°C and further release of surface hydroxyl groups between 200 and 400°C. Between 200 and 500 °C organic surface groups such as acetate in the case of TiO_2 and glutaraldehyde in the case of SiO_2 decompose and carbon-based gases evolve. After the photocatalytic batch-mode run, more adsorbed water is found in all spent catalyst samples and slight changes in the evolving gas emissions of the carbon-based organic and hydroxylic surface groups are detected. The photocatalytic H_2O_2 production of the studied catalysts is maintained during a second catalytic run, indicating the physical and chemical stability of the supraparticles.

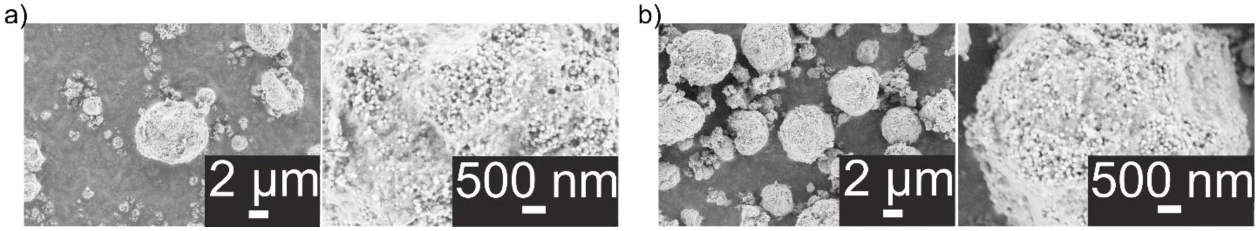

Figure S18. SEM overview micrographs (on the left) and close-up views of spray-dried supraparticles consisting of TiO_2 and SiO_2 nanoparticles in a weight ratio of 1:2 before (a) and after the continuous flow reaction (b).

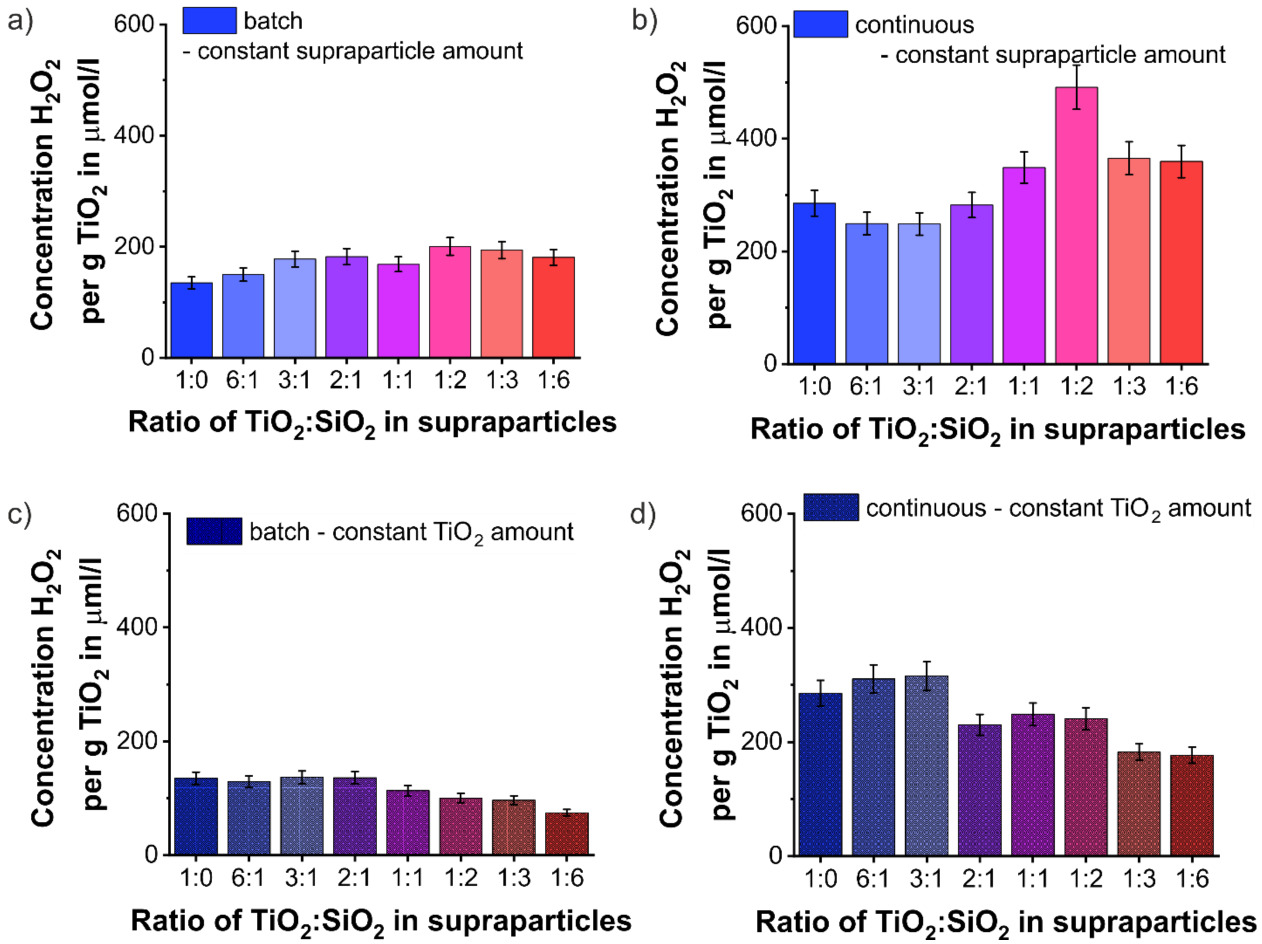

Figure S19. Cumulated concentration of H_2O_2 after 5 min UV-light exposure produced by core-shell supraparticles with increasing SiO_2 nanoparticle contents, *i.e.* rising from a TiO_2 to SiO_2 weight ratio of 6:1 to 1:6, in comparison to pure TiO_2 supraparticles (1:0 TiO_2:SiO_2 ratio) referenced to the weight of contained photocatalyst within each supraparticle sample: obtained in a) batch reactions and b) continuous flow reactions keeping the total supraparticle amount per batch constant, as well as in c) batch reactions and d) continuous flow reactions keeping the total TiO_2 photocatalyst amount per batch constant.

References

(1) Tsukamoto, D.; Shiro, A.; Shiraishi, Y.; Sugano, Y.; Ichikawa, S.; Tanaka, S.; Hirai, T. Photocatalytic H 2 O 2 Production from Ethanol/O 2 System Using TiO 2 Loaded with Au–Ag Bimetallic Alloy Nanoparticles. *ACS Catal.* **2012**, *2*, 599–603.

(2) Maurino, V.; Minero, C.; Pelizzetti, E.; Mariella, G.; Arbezzano, A.; Rubertelli, F. Influence of Zn(II) adsorption on the photocatalytic activity and the production of H2O2 over irradiated TiO2. *Res. Chem. Intermed.* **2007**, *33*, 319–332.

(3) Cai, R.; Kubota, Y.; Fujishima, A. Effect of copper ions on the formation of hydrogen peroxide from photocatalytic titanium dioxide particles. *Journal of Catalysis* **2003**, *219*, 214–218.

(4) Teranishi, M.; Naya, S.; Tada, H. In situ liquid phase synthesis of hydrogen peroxide from molecular oxygen using gold nanoparticle-loaded titanium(IV) dioxide photocatalyst. *Journal of the American Chemical Society* **2010**, *132*, 7850–7851.

(5) Zhao, Y.; Kondo, Y.; Kuwahara, Y.; Mori, K.; Yamashita, H. Hydrophobic and visible-light responsive TiO2 as an efficient photocatalyst for promoting hydrogen peroxide production in a two-phase system. *Catalysis Today* **2024**, *425*, 114350.
